# Cortical morphology at birth reflects spatiotemporal patterns of gene expression in the fetal human brain

**DOI:** 10.1371/journal.pbio.3000976

**Published:** 2020-11-23

**Authors:** Gareth Ball, Jakob Seidlitz, Jonathan O’Muircheartaigh, Ralica Dimitrova, Daphna Fenchel, Antonios Makropoulos, Daan Christiaens, Andreas Schuh, Jonathan Passerat-Palmbach, Jana Hutter, Lucilio Cordero-Grande, Emer Hughes, Anthony Price, Jo V. Hajnal, Daniel Rueckert, Emma C. Robinson, A David Edwards

**Affiliations:** 1 Developmental Imaging, Murdoch Children’s Research Institute, Melbourne, Australia; 2 Centre for the Developing Brain, Department of Perinatal Imaging & Health, King’s College London, United Kingdom; 3 Department of Paediatrics, University of Melbourne, Melbourne, Australia; 4 Developmental Neurogenomics Unit, National Institute of Mental Health, Bethesda, United States of America; 5 Department of Psychiatry, University of Cambridge, United Kingdom; 6 Department of Electrical Engineering, ESAT/PSI, KU Leuven, Belgium; 7 Biomedical Image Analysis Group, Department of Computing, Imperial College London, United Kingdom; University Medical Center Eppendorf, Hamburg University, GERMANY

## Abstract

Interruption to gestation through preterm birth can significantly impact cortical development and have long-lasting adverse effects on neurodevelopmental outcome. We compared cortical morphology captured by high-resolution, multimodal magnetic resonance imaging (MRI) in *n* = 292 healthy newborn infants (mean age at birth = 39.9 weeks) with regional patterns of gene expression in the fetal cortex across gestation (*n* = 156 samples from 16 brains, aged 12 to 37 postconceptional weeks [pcw]). We tested the hypothesis that noninvasive measures of cortical structure at birth mirror areal differences in cortical gene expression across gestation, and in a cohort of *n* = 64 preterm infants (mean age at birth = 32.0 weeks), we tested whether cortical alterations observed after preterm birth were associated with altered gene expression in specific developmental cell populations. Neonatal cortical structure was aligned to differential patterns of cell-specific gene expression in the fetal cortex. Principal component analysis (PCA) of 6 measures of cortical morphology and microstructure showed that cortical regions were ordered along a principal axis, with primary cortex clearly separated from heteromodal cortex. This axis was correlated with estimated tissue maturity, indexed by differential expression of genes expressed by progenitor cells and neurons, and engaged in stem cell differentiation, neuron migration, and forebrain development. Preterm birth was associated with altered regional MRI metrics and patterns of differential gene expression in glial cell populations. The spatial patterning of gene expression in the developing cortex was thus mirrored by regional variation in cortical morphology and microstructure at term, and this was disrupted by preterm birth. This work provides a framework to link molecular mechanisms to noninvasive measures of cortical development in early life and highlights novel pathways to injury in neonatal populations at increased risk of neurodevelopmental disorder.

## Abstract 2

Interruption to gestation through preterm birth can significantly impact cortical development and have long-lasting adverse effects on neurodevelopmental outcome. A large neuroimaging study of newborn infants reveals how their cortical structure at birth is associated with patterns of gene expression in the fetal cortex and how this relationship is affected by preterm birth.

## Introduction

The mammalian cortex is composed of functionally distinct regions organised along broad gradients that reflect spatially ordered and concerted variations of cortical structure and function [1–6]. While the mechanisms behind the emergence of this complex topography are not fully understood, cortical patterning is underwritten by dynamic regulation of gene transcription during gestation [7,8]. During embryonic development, early patterning of the neuroepithelium is established though intrinsic genetic mechanisms [9–12] that regulate early neurodevelopmental processes including neurogenesis and neuronal migrations from around 6 to 8 postconceptional weeks (pcw) in humans [11,13]. During fetal development, this leads to the establishment and expansion of transient neural structures, including the subventricular zone, preplate, and subplate, and, eventually, formation of the cortex [11,14,15].

The advent of modern transcriptomic technologies has allowed the precise mapping of cortical gene expression during the human fetal period [16–18]. Gene transcription is highly differentially expressed during prenatal development and varies significantly across cortical areas [8,16,17,19]. Interruption to the precisely timed dynamics of gene transcription during gestation is implicated in the onset of common developmental cognitive and neuropsychiatric disorders [18,20,21].

Recently, the postmortem transcription of thousands of genes across the adult brain has been compiled to form brain-wide gene expression atlases [18,22,23]. This allows precise comparison between spatial patterns of cortical gene expression and neuroanatomy quantified using magnetic resonance imaging (MRI) [24]. Neuroimaging studies have found that patterns of gene expression in the adult cortex are mirrored by regional variation in cortical morphometry [25] and functional organisation [26] and are associated with neuroimaging markers of developmental disorders [27]. Similar databases detailing cerebral gene transcription across the full human life span from early embryonic stages to adulthood are now available [16,18]. This has created an unprecedented opportunity to explore the molecular correlates of neuroimaging markers of early brain development.

Advances in neonatal neuroimaging now permit the quantification of developmental neuroanatomy in vivo at a higher resolution than previously possible [28,29]. Imaging studies of the developing human brain shortly after birth have characterised a highly dynamic period of cerebral change defined by significant increases in brain volume [30,31], cortical thickness and surface area [31–33], progressive white matter myelination [34,35], and ongoing configuration and consolidation of functional brain networks [36–41]. Several studies have also used diffusion MRI models to study the microstructure of the cortex at around the time of birth, identifying areal patterns of development that may relate to ongoing cellular processes including dendritic arborisation and synaptic formation [42–46]. Further, the truncation of gestation due to preterm birth is associated with widespread alterations in cortical morphometry and microstructure indexed by MRI at the time of normal birth that highlight the sensitivity of noninvasive neuroimaging to detect disruptions in early developmental processes [32,42–49].

The combination of these technologies opens a new window to study early human brain development, facilitating a comparison between patterns of prenatal cortical gene expression and the development of the brain at around the time of birth, as well as providing a platform to test mechanistic hypotheses about the impact of early disruptions to brain development during gestation. The potential of this approach has been previously demonstrated using postmortem MRI to reveal a correspondence between genes linked to neural development and the microstructure of the fetal cortex [50]. In this study, we explore the association between in vivo measures of cortical morphometry at birth and regional patterns of fetal gene transcription in the human brain. We test the hypothesis that noninvasive markers of neonatal cortical structure mirror areal differences in the timing of cellular processes underlying cortical development, as indexed by differential spatiotemporal patterning of gene expression in the fetal cortex. Additionally, we test whether cortical alterations observed after preterm birth and quantified with MRI are linked with a selective vulnerability of developmental neuronal and glial cell populations in the developing cortex.

We define a principal mode of variation in neonatal cortical structure that is aligned to differential patterns of genes expression in the fetal cortex, enriched for foundational neurodevelopmental processes, including neuronal differentiation and migration, and disrupted by preterm birth.

## Results

### A principal axis of the neonatal cortex

**Fig 1:**
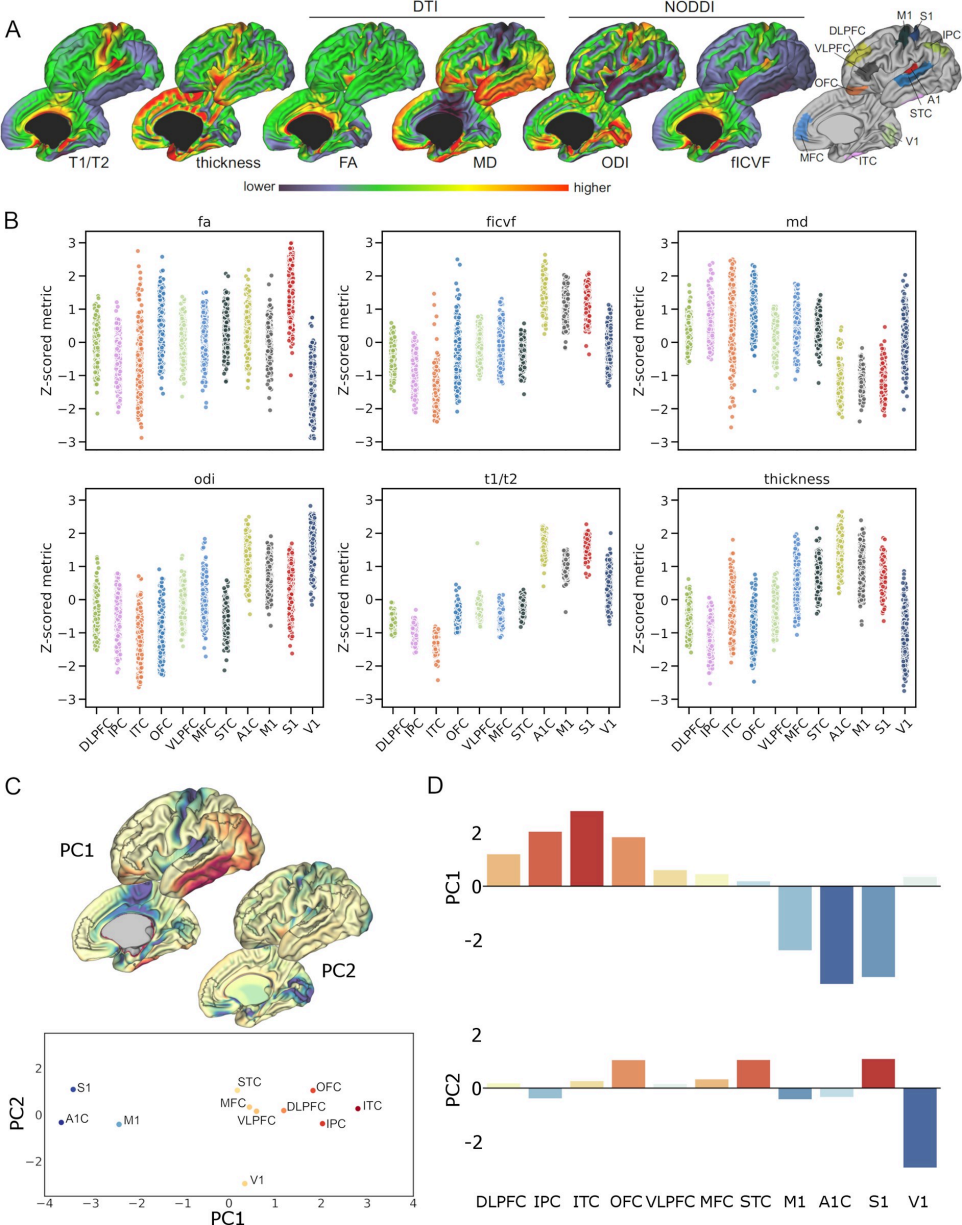
A principal axis of the neonatal cortex indexed by multimodal MRI. (A) Average cortical neuroimaging metrics in a cohort of healthy, term-born neonates (*n* = 292). Metrics derived from structural MRI (T1/T2 contrast and cortical thickness) and diffusion MRI model parameters using DTI (FA and MD) and NODDI (fICVF and ODI). Right: cortical ROI based on anatomical references with corresponding developmental transcriptomic data (S1 Fig). (B) Z-scored cortical metrics are shown for each participant grouped within each cortical ROI. (C) Top: cortical representations of the first 2 principal components (PC1 and PC2) derived from the PCA of the regional MRI metric data in B. Bottom: position of each cortical ROI in PCA state space; the position of each region is dictated by its component score (D) for the first 2 principal components. Regions are labelled and coloured by PC1 score. (D) PCA scores of each metric for the first 2 principal components, coloured by PC1 score. See https://github.com/garedaba/baby-brains/tree/master/figures. A1, primary auditory cortex; DLPFC, dorsolateral prefrontal cortex; DTI, diffusion tensor imaging; FA, fractional anisotropy; fICVF, intracellular volume fraction; IPC, inferior parietal cortex; ITC, inferior temporal cortex; M1, primary motor cortex; MD, mean diffusivity; MFC, medial frontal cortex; MRI, magnetic resonance imaging; NODDI, neurite orientation dispersion and density imaging; ODI, orientation dispersion index; OFC, orbitofrontal cortex; PCA, principal component analysis; ROI, regions of interest; S1, primary sensory cortex; STC, superior temporal cortex; V1, primary visual cortex; VLPFC, ventrolateral prefrontal cortex.

Using high-resolution structural and diffusion MRI data acquired from a large cohort of healthy neonates (*n* = 292, 54% male, median [range] gestational age at scan = 40.86 [37.29 to 44.71]), we extracted 6 measures of cortical morphology (cortical thickness) and microstructure (T1w/T2w contrast, fractional anisotropy [FA], mean diffusivity [MD], intracellular volume fraction [fICVF], and orientation dispersion index [ODI]) from 11 cortical regions of interest (ROI) with corresponding mRNA sequencing (mRNA-seq) in a prenatal transcriptomic dataset [18] (Fig 1A and S1 Fig).

Normalised regional metrics for all participants are shown in Fig 1B. Similar regional profiles are evident across metrics with the pattern of interregional variation reflecting the full transcortical patterns shown in Fig 1A. Comparable regional patterns were observed in FA and cortical thickness and between ODI, fICVF, and the T1w/T2w contrast (Fig 1B), with higher FA, thicker cortex, and a higher T1w/T2w contrast in primary somatomotor cortex (Fig 1A and 1B). MD displayed an opposing trend across regions, lowest in primary somatomotor regions and highest in frontoparietal regions.

Based on the similarities in cortical patterning across metrics, we hypothesised that regional variation across metrics could be represented by a small number of latent factors. Using principal component analysis (PCA), we projected the regional metrics onto a set of principal axes that maximally explained variance in the full set of cortical measures (Fig 1C). Using the group average region × metric matrix, we found that the first 2 components explained 91.6% of the total variance (PC1 = 72.3% and PC2 = 19.3%, respectively).

The ordering of regions along the principal axis (PC1; Fig 1C) illustrates a clear separation between primary and higher-order cortical regions based on neuroimaging metrics, with primary somatosensory and motor cortex (primary auditory cortex [A1C], primary motor cortex [M1], and primary sensory cortex [S1]) situated at opposite ends to prefrontal, inferior parietal, and temporal cortex (dorsolateral prefrontal cortex [DLPFC], inferior parietal cortex [IPC], and inferior temporal cortex [ITC]). This pattern is apparent in all cortical metrics, most strongly in T1/T2 contrast, fICVF, and MD (S2 Fig). The second principal axis (PC2) predominantly captured anatomical and microstructural differences in the primary visual cortex (V1) compared to other primary cortex (Fig 1C and 1D).

### PC1 is associated with regional patterns of gene expression in mid-gestation

Using a developmental transcriptomic dataset of bulk tissue mRNA data sampled from cortical tissue in 16 prenatal human specimens [18], we compared regional variation in cortical MRI metrics, represented by PC1, with prenatal gene expression in anatomically correspondent cortical regions. Through comparison to 5 independent single-cell RNA studies of the developing fetal cortex [18,51–54], we selected a set of 5,287 marker genes shown to be differentially expressed in cortical cell populations during gestation. We used a nonlinear mixed-effects approach to model developmental changes in gene expression in reads per kilobase of transcript per million mapped reads (RPKM) as a smooth function of age, accounting for inter-specimen variability. The nonlinear model provided a better fit of the expression data for all genes compared to a comparable linear model (range Akaike information criterion [AIC] difference: −16.7 to −87.7; range Bayesian information criterion [BIC] difference: −2.1 to −58.9).

Using specimen- and age-corrected RPKM values provided by the residuals of the nonlinear mixed model for each gene (S3 Fig), we tested the association between spatial variation in gene expression during gestation and regional PC1 score using nonparametric correlation (Kendall’s *τ*). Of 5,287 genes, 120 displayed a significant (positive or negative) correlation with PC1 after correction for multiple comparisons with false discovery rate (FDR; *p* < 0.05). In total, 71 genes were positively correlated with PC1, with increasing gene expression in regions with a higher PC1 score (mean ± SD *τ* = 0.208 ± 0.023) and 49 genes displayed the opposite relationship, with higher expression in regions with a negative PC1 score (mean ± SD *τ* = −0.208 ± 0.022).

**Fig 2:**
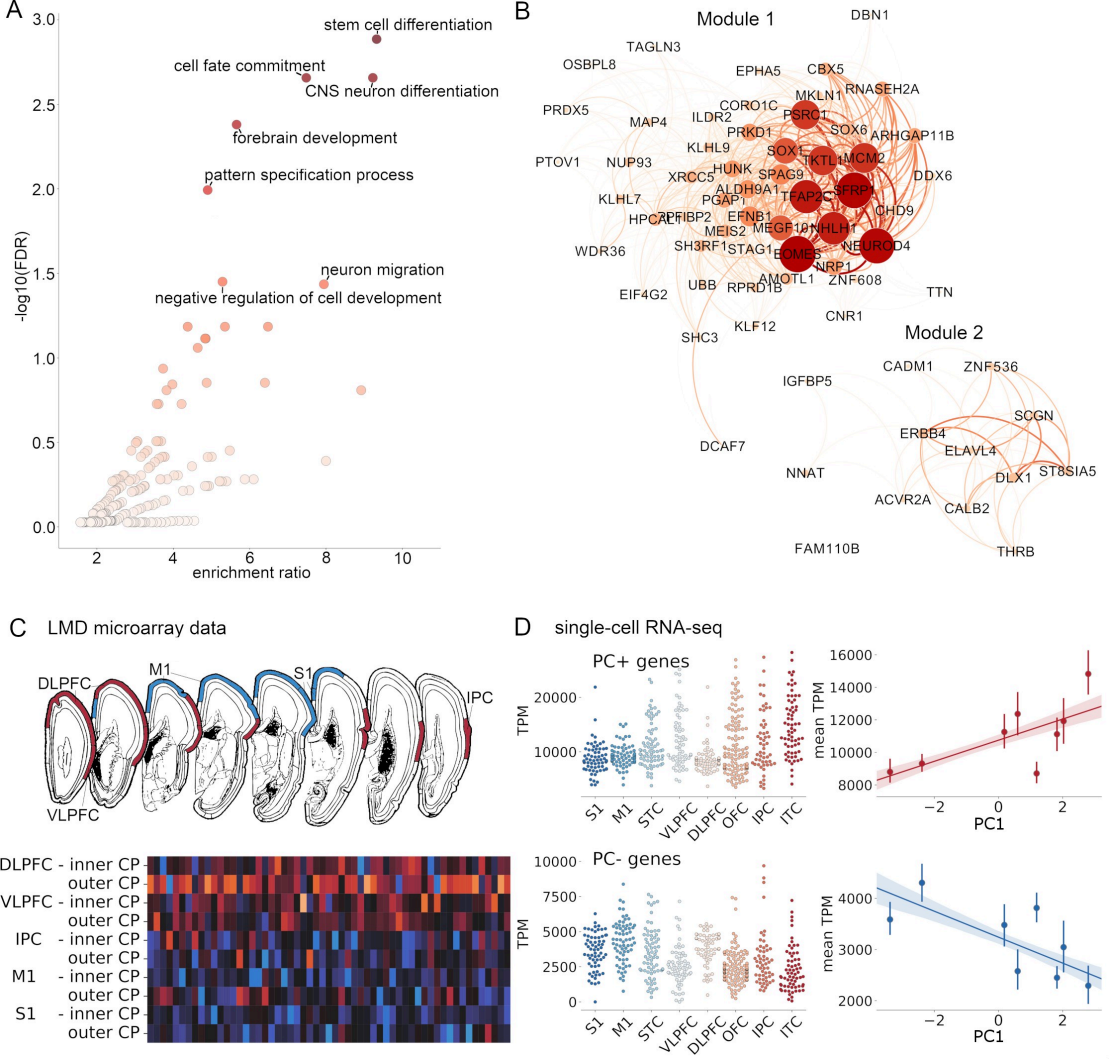
Genes associated with neuronal differentiation are differentially expressed along the principal imaging axis. (A) Volcano plot showing enrichment of GO terms (biological processes) in genes with age-corrected expression levels positively correlated with PC1. Significantly enriched terms (FDR < 0.05, reference: protein-coding genes) are labelled. (B) Gene co-expression analysis of all PC+ genes revealed 2 modules). Intra-modular connections are shown with node size and colour indicating strength and edge thickness and colour indicating weight. (C) Differential expression of PC+ genes across cortical regions (top) measured using LMD microarrays of the cortical plate in two 21-pcw fetal samples (https://www.brainspan.org/lcm/). Heatmap shows relative expression of all 71 PC+ genes in the inner and outer cortical plate of each labelled region. (D) Total expression (in TPM) of PC+ (top) and PC− (bottom) genes in single cells (*n* = 572) extracted from cortical regions in an independent single-cell RNA-seq survey of the mid-gestational fetal cortex [51]. Scatterplots show mean TPM averaged over cells in each region, correlated with each region’s PC1 score. See https://github.com/garedaba/baby-brains/tree/master/figures and https://github.com/garedaba/baby-brains/tree/master/results/wgcna. CNS, central nervous system; DLPFC, dorsolateral prefrontal cortex; FDR, false discovery rate; GO, Gene Ontology; IPC, inferior parietal cortex; ITC, inferior temporal cortex; LMD, laser microdissection; M1, primary motor cortex; OFC, orbitofrontal cortex; RNA-seq, RNA sequencing; S1, primary sensory cortex; STC, superior temporal cortex; TPM, transcripts per million; VLPFC, ventrolateral prefrontal cortex.

We reasoned that genes associated with the patterning of cortical morphometry at birth may subserve important neurodevelopmental functions. To test this, we performed an overrepresentation analysis (ORA) [55] for ontological terms associated with specific biological processes in both gene lists. Of 71 genes with spatial patterns of expression positively correlated with PC1 (denoted PC+), 61 (86%) were annotated to specific functional terms. Using all protein-coding genes transcribed in the bulk RNA dataset as the background reference set, we found significant enrichment of several neurodevelopmental terms including stem cell differentiation (FDR = 0.001, enrichment ratio = 9.32), neuron migration (FDR = 0.03, enrichment = 7.94), and forebrain development (FDR = 0.004, enrichment = 5.65) (Fig 2A and S1 Table). Terms relating to stem cell and neuronal differentiation remained significantly enriched when restricting the background reference set to only include genetic markers of fetal cortical cells (*n* = 5,287; S1 Table). Performing weighted gene correlation network analysis (WGCNA) on the PC+ gene set, we identified 2 co-expression modules (Fig 2B). The largest contained 53 genes including a tightly correlated set of developmental genes with roles in regulating cell growth and differentiation including *EOMES*, *NEUROD4*, *SFRP1*, and *TFAP2C*. The smaller second module (Module 2) contained 13 genes, with roles including neuronal signalling (*ERBB4*, *CALB2*, and *SCGN*) and neuronal differentiation (*ZNF536* and *DLX1*).

No biological terms were significantly enriched in genes with a spatial pattern of expression negatively correlated with PC1 (denoted PC−). Using WGCNA, 3 small modules of 7 genes each were identified (Modules 1N to 3N; S4 Fig), including genes with high neuronal expression (Module 1N; *CDKL5*, *ZBTB18*, and *SORCS1*) and genes involved in cellular processes including adhesion and signalling (Module 2N: *ACTN2*, *PTPN2*, and *SSX2IP*) and metabolic activity (Module 3N: *DUSP7* and *ST3GAL1*).

Using independent microarray data from laser microdissection (LMD) of the 21-pcw fetal cortex [16], we verified that PC+ genes had higher expression in the cortical plate of higher-order regions (DLPFC, ventrolateral prefrontal cortex [VLPFC], and IPC) compared to primary cortex (M1 and S1) (Fig 2C; mean fold change = 1.36, *p* < 0.001, 10,000 permutations). Using the top 100 differentially expressed genes (DEGs) identified in the LMD dataset, we also confirmed that genes with higher expression in regions with a higher PC1 score (DLPFC, VLPFC, and IPC) in mid-gestation were enriched for important neurodevelopment functions including neuron differentiation (Gene Ontology [GO]: 0021953, FDR = 0.019, reference: all genes; S2 Table). Additional validation experiments using independent single-cell RNA sequencing (RNA-seq) data [51] confirmed an association between regional PC1 score at birth and expression of PC1+ and PC− gene sets in mid-gestation (Fig 2D).

### Imaging–gene associations are enriched for specific cell types in the fetal cortex

To explore these relationships further, we reconstructed cellular gene expression profiles by stratifying the bulk tissue expression data using genetic markers of cell type derived from single-cell RNA studies of the fetal cortex [18,51–54].

**Fig 3:**
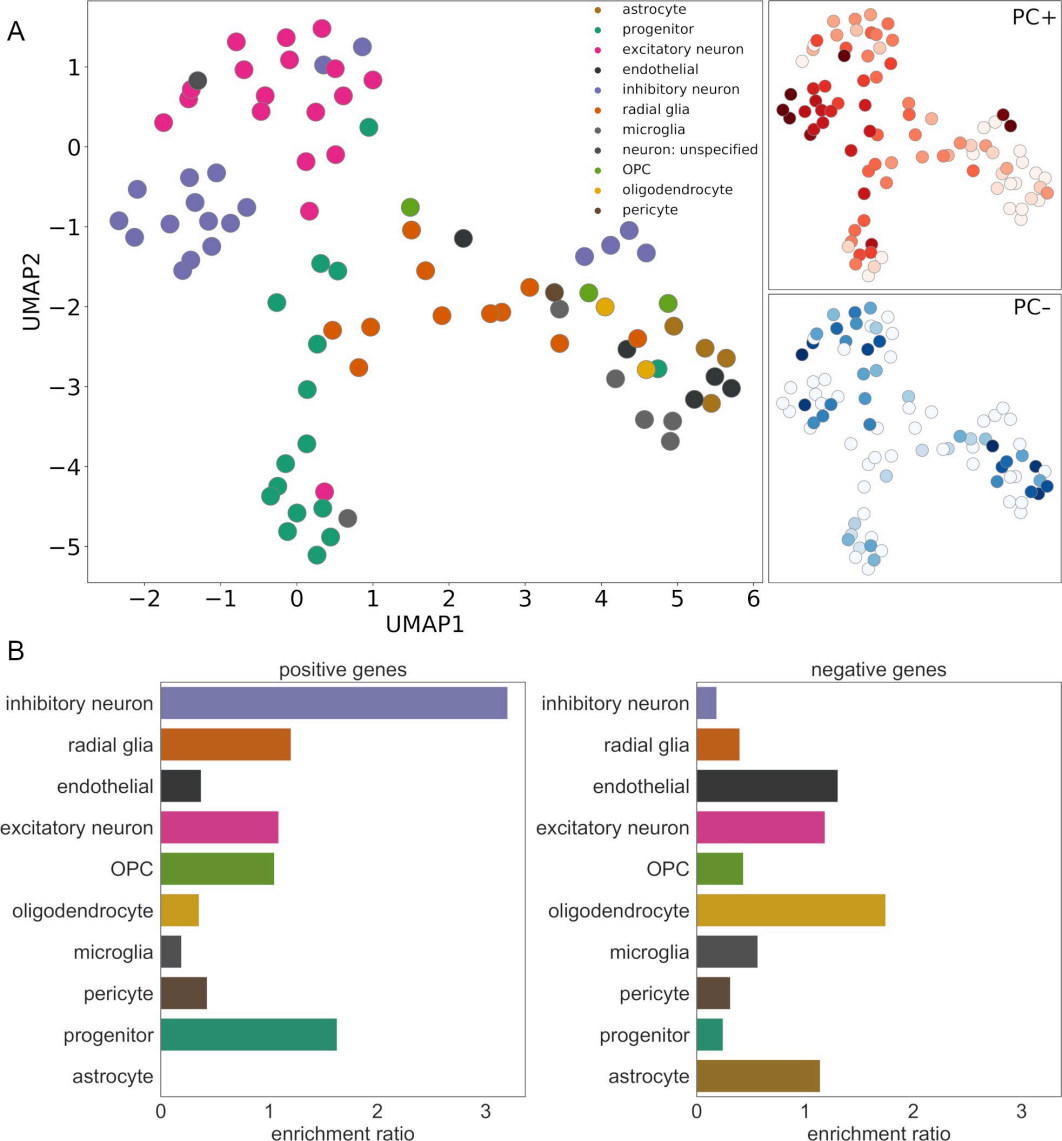
Cell-specific gene expression is associated with cortical morphology at birth. (A) UMAP embedding of 86 cell types based on trajectories of relative gene expression over time recovers annotated cell classes. Subplots reflect enrichment ratios of cell classes in PC+ and PC− gene sets (darker colour represents higher enrichment ratio). (B) Enrichment ratio for fetal marker genes expressed by each cell class is shown for PC+ (left) and PC− (right) gene sets. See https://github.com/garedaba/baby-brains/tree/master/figures. OPC, oligodendrocyte precursor cell; UMAP, Uniform Manifold Approximation and Projection.

Sets of genetic markers for 11 cortical cell classes were initially compiled by combining lists of genes that are differentially expressed in fetal cortical cell populations (S3 Table). To verify this grouping, we calculated the average expression trajectories for all genetic markers within each cell type across gestation and used them to calculate a 2D embedding using Uniform Manifold Approximation and Projection (UMAP; Fig 3A). Proximity in the embedded space reflects similarity between average trajectories of gene expression within cell type over time. In the embedded space, cell types clustered by assigned class, and maturational timing (e.g., precursor or mature), as well as within cellular subtype (e.g., inhibitory and excitatory neurons; S5 Fig).

We tested the enrichment of genes expressed by each cell class within the PC+ and PC− gene sets. We found that PC+ genes were significantly enriched for genes expressed by precursor cells (*p* = 0.0003, reference: fetal gene markers), specifically, for genes expressed by intermediate progenitor cells (enrichment ratio = 1.63, *p* = 0.0002; S4 Table) and inhibitory neurons (enrichment ratio = 3.2, *p* < 0.0001; Fig 3B and S4 Table). Post hoc analysis within cell class revealed specific inhibitory neuron subtypes present in the mid-fetal brain and enriched in the PC+ gene set included migrating cortical interneurons from the caudal ganglionic eminence (In_5 [51] and IN-CTX-CGE2 [52] both *p* < 0.0001) and newborn interneurons originating in the medial ganglionic eminence (nIN1; [52] *p* = 0.0017).

In contrast, PC− genes, with a spatial pattern of expression that was higher in primary somatomotor regions at mid-gestation, were enriched for genes expressed by mature cell types (enrichment = 1.18, *p* = 0.002). In terms of cell class, genes expressed by oligodendrocytes were enriched within PC−, though not significantly (enrichment = 1.75, *p* = 0.056; Fig 3B and S4 Table). When considering only marker genes uniquely expressed by each cell class, PC− genes were enriched for excitatory neuronal genes (enrichment = 2.14, *p* = 0.008; S5 Table). Post hoc analysis within this class revealed a single enriched early-maturing excitatory neuronal subtype (Ex_4 [51], *p* < 0.0001). Similar patterns of cell class–specific expression of PC+ and PC− genes were observed in the single-cell RNA dataset (S6 Fig).

### Variation in tissue maturation during gestation predicts cortical development at birth

These data suggest that the spatial patterning of gene expression in the developing cortex is mirrored by regional variation in cortical morphology and microstructure measured using MRI at birth. To test this hypothesis, we created a model of cortical maturity to capture the relationship between the regional timing of gene expression and tissue maturation.

**Fig 4:**
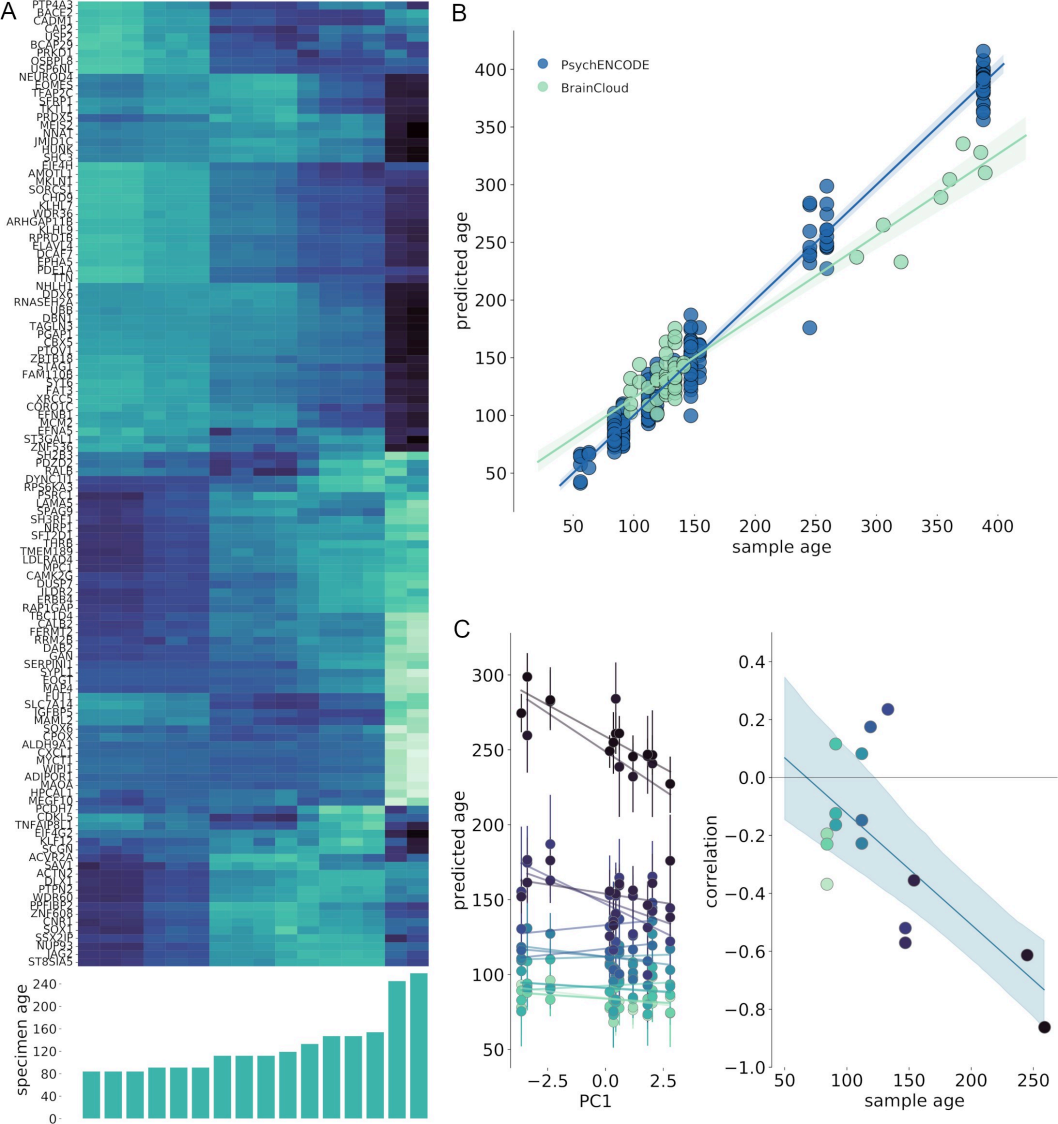
Tissue maturity correlates with regional variation in cortical morphometry at birth. (A) Developmental patterns of mean cortical gene expression illustrated in each specimen for all 120 regionally variant genes (PC+ and PC−), ordered by age. (B) The relationship between predicted and true sample age for all regional samples (*n* = 198 samples from *n* = 21 brains) in the PsychENCODE dataset aged between 50 and 400 postconceptional days (8 pcw to 4 postnatal months), estimated using SVR and LOO cross-validation. The SVR model was validated using additional samples from the BrainCloud dataset (*n* = 46 samples). Shaded area indicates 95% CI. (C) Left: The correlation between regional PC1 score and predicted tissue maturity is shown for each sample during gestation. Error bars show 95% CI for regional age predictions over 1,000 bootstrapped gene samples. Right: PC1 correlation is plotted against specimen age for each brain. Shaded area indicates 95% CI for linear model fit over bootstrap samples. See https://github.com/garedaba/baby-brains/tree/master/figures. CI, confidence interval; LOO, leave-one-out; SVR, support vector regression.

We modelled tissue sample age as a function of gene expression using support vector regression (SVR). To ensure full coverage across the prenatal period and to maximise the number of samples contributing to the model, we included additional data from all tissue samples from brains aged 8 pcw to 4 months postnatal age (*n* = 21 total). Using mean cortical gene expression of all 120 (PC+ and PC−) genes (Fig 4A), our model accurately predicted sample age across the full prenatal window (Fig 4B), up to 4 months of age. We validated our model in a separate dataset comprising microarray data from the prefrontal cortex in *n* = 46 brains aged 13 pcw to 4 months [56] (BrainCloud; Fig 4B).

Using predictions from this model, we estimated the correlation between regional age predictions and PC1 in the prenatal sample (Fig 4C, left). We expected that for a given brain, regions with a more advanced gene expression profile (i.e., more similar to older tissue samples) would return an older age prediction. We observed a negative association develop over gestation between a cortical region’s predicted maturity and its position along the principal axis at birth (Fig 4C, right; r^2^ = 0.36, *p* < 0.001 [5,000 permutations]), such that in older samples, a lower PC1 score was associated with an older predicted age based on gene expression.

Using nonlinear models of gene expression over time, we estimated regional genetic maturity at several points across gestation (S7 Fig). We found that the relative maturity of regions compared to the rest of the cortex varied over time. Primary somatomotor regions remained relatively advanced throughout gestation compared to the rest of the cortex. In contrast, V1 remained relatively delayed across gestation. A divergence in maturity becomes apparent within higher-order regions by mid-gestation, with some cortical areas (IPC and ITC) falling behind other regions towards the time of birth. These patterns were largely repeated using the full fetal gene marker set (*n* = 5,287 genes; S8 Fig).

### Preterm birth leads to alterations along the principal imaging axis

Based on this evidence, we hypothesised that an interruption to the length of gestation would yield differences in cortical morphology indexed by variation along PC1. To test this, we compared cortical morphology in healthy neonates (*n* = 292) to a cohort of preterm-born infants scanned at term-equivalent age (*n* = 64, 59% male; mean [SD] gestational age at birth = 32.00 [3.88] weeks).

**Fig 5:**
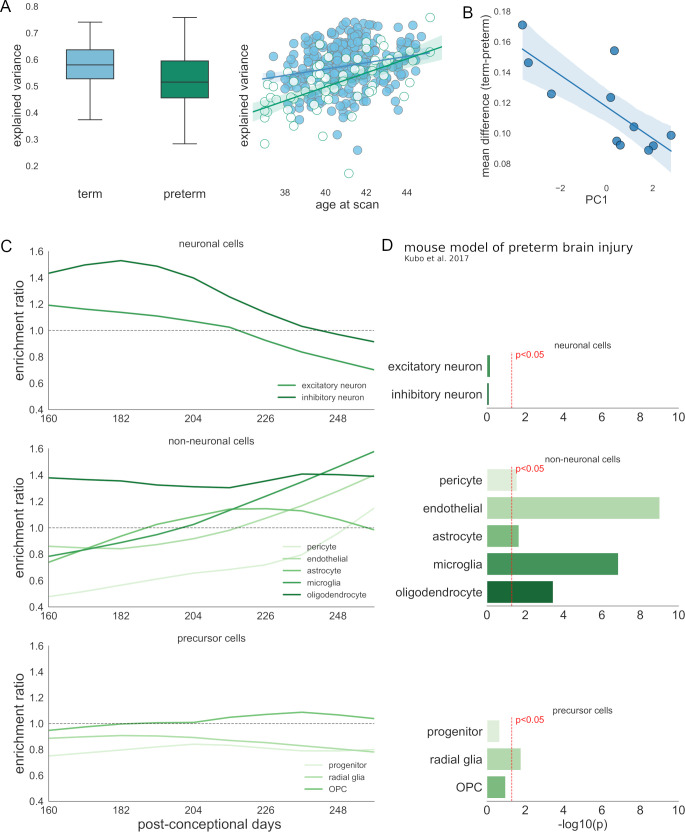
Disruption of cortical development in preterm-born infants. (A) Left: group difference in individual variance across multiple neuroimaging metrics explained by the principal imaging axis in term (blue) and preterm (green) infants (left). Right: the relationship between age at scan and variance explained by PC1 across all cortical metrics (right). Regression lines are shown for term (blue) and preterm (green) infants with 95% CI. (B) Group differences in regional T1w/T2w contrast ordered by position along PC1 (linear regression shown with 95% CI). (C) Enrichment of gene sets from 10 fetal cortical cell classes (top: neuronal; middle: nonneuronal; bottom: precursor) based on genes significantly associated (FDR *p* < 0.05) with group differences in T1w/T2w contrast at 10 time points in the preterm period. (D) Enrichment of genes both expressed by each cell type and significantly correlated with T1/T2 in at least 1 age window in DEGs measured in an experimental mouse model of preterm brain injury [57]. See https://github.com/garedaba/baby-brains/tree/master/figures. CI, confidence interval; DEG, differentially expressed gene; FDR, false discovery rate.

We extracted neuroimaging metrics from each cortical region and projected each individual’s region × metric matrix onto the principal imaging axis (S9 Fig). After correcting for age at scan and sex, regional variation along PC1 explained significantly less variance in preterm individual’s imaging data than those born at term (ANCOVA: F = 7.9, *p* = 0.005; Fig 5A). Across both groups, the mean variance explained by PC1 increased with age (Fig 5A; F = 46.0, *p* < 0.001), with a stronger association in the preterm cohort (interaction: F = 6.63, *p* = 0.01), suggesting that arrangement along the principal axis is ongoing around the time of birth and altered by events surrounding preterm birth. There was no significant difference between sexes (F = 1.12, *p* = 0.28).

As differences in the variance explained by PC1 are dictated by individual differences in cortical metrics, we sought to test the specific effects of preterm birth on all imaging measures. Using mixed effects linear models including effects of age, birth status, and regional PC1 score, we confirmed a significant main effect of birth status on all cortical metrics except for ODI (S6–S8 Tables). The largest effect was evident in cortical T1w/T2w contrast (F_1,354_ = 135.53, *p* < 0.0001, Cohen’s *d* = 1.62; S8 Table). On average, cortical T1w/T2w was significantly lower in preterm infants (marginal means [95% CI] = 1.32 [1.31, 1.33], 1.20 [1.18, 1.22] for term and preterm infants, respectively). To a lesser extent, both fICVF and FA were, on average, higher in term infants (*d* = 0.32, 0.56, respectively), although the direction of this effect was not consistent across cortical regions (S10 Fig). In contrast, average cortical MD (*d* = −1.17) and, to a lesser extent, cortical thickness (*d* = −0.65) were higher in preterm infants across all regions.

The magnitude of regional group differences across all cortical metrics varied as a function of PC1 (Fig 5B, S7 Table and S10 Fig). This effect was most apparent in T1w/T2w contrast where the differences between term and preterm groups formed a strong negative association with PC1 (r = −0.78, *p* = 0.023 after FDR correction). Similar trends were seen in the other metrics, although none reached significance (|r| = 0.32 to 0.68, all *p* > 0.05).

### Vulnerability of specific cell populations to the timing of preterm birth

These data show that cortical differences in preterm infants occur along the principal imaging axis and are most apparent in T1/T2w contrast. We investigated the potential that the differences observed in preterm cortex may reflect a selective vulnerability in specific cell populations due to coincidental timing of extrauterine exposure following preterm birth and temporal variations in gene expression. Focusing on the cortical differences observed in T1/T2w contrast, we first estimated gene expression trajectories over the latter stages of gestation (160 to 260 postconceptional days, approximately 25 to 39 weeks gestational weeks). We then split this period into 10 age windows, and within each, we identified genes with expression significantly correlated with the magnitude of group differences in T1/T2w contrast at term-equivalent age (FDR-corrected *p* < 0.05, Fig 5C). Within each window, we tested for enrichment of gene expression by each of 10 fetal cell types. In the early preterm period, we found that mean regional differences in T1w/T2w contrast at term-equivalent age were significantly associated with genes expressed by both inhibitory and excitatory neurons (windows 1, 2, 3, and 5, hypergeometric statistic: *p* < 0.05; Fig 5C, top; reference: fetal cell markers). However, later in gestation, T1w/T2w differences were correlated with the expression of genes enriched for glial cell populations, including microglia, endothelial cells (windows 8, 9, and 10; all *p* < 0.05), and oligodendrocytes (windows 1, 2, 8, 9, and 10, all *p* < 0.05; Fig 5C, middle).

Using gene expression data from a mouse model of preterm brain injury [57], we confirmed the relationship between preterm brain injury and altered gene expression in glial cell populations at birth (Fig 5D). Gene expression in the murine cortex was measured at P1.5, after hypoxic–ischemic insult at E16.5. DEGs (*p* < 0.05) were mapped to human homologs with 217 DEGs matched to human genes included in the current study. We found that the set of DEGs was enriched for genes both expressed by glial populations in the human fetal cortex and associated with T1/T2 differences in the neonatal cortex (Fig 5D and S9 Table). Relative to a background set of mapped genes (*n* = 15,052) we observed a significant enrichment of genes associated with T1/T2 in at least 1 age window and expressed by glial populations including microglia (enrichment ratio = 6.9, *p* < 0.001), endothelial cells (5.4, *p* < 0.001), oligodendrocytes (4.9, *p* < 0.001), and radial glia (2.2, *p* = 0.02). These relationships remained significant in microglia and endothelial cells when restricting the background set to only include matched fetal gene markers (*n* = 4,733).

### Potential cellular processes disrupted in the preterm brain

**Fig 6:**
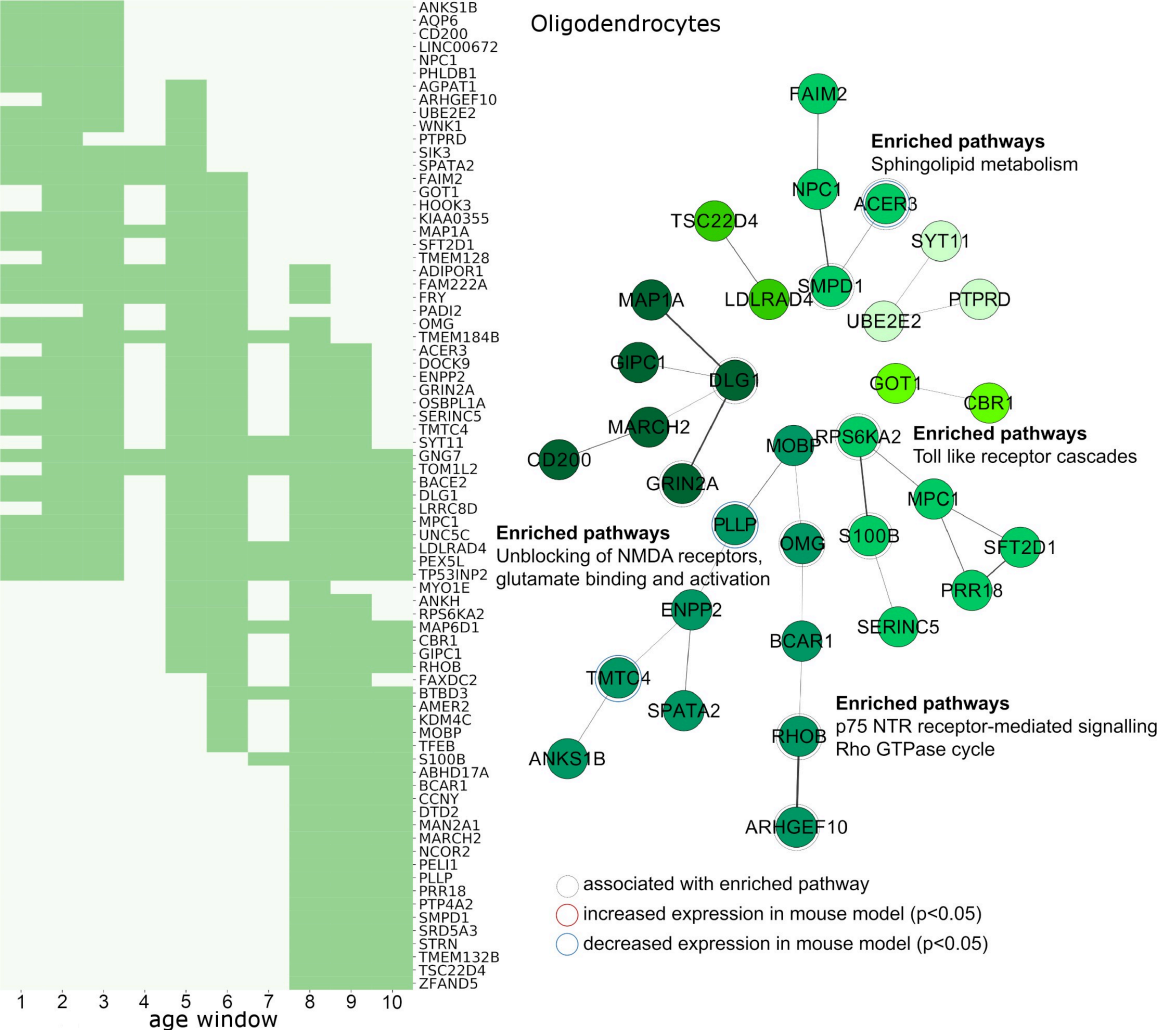
Cellular pathways associated with genes expressed by oligodendrocytes and developmental alterations in the preterm cortex. Left: Genes expressed by oligodendrocytes in the fetal cortex and significantly associated with group differences in T1w/T2w contrasts across at least 3 age windows are shown. Dark green indicates periods where gene expression and T1w/T2w contrast were significantly correlated for each gene (FDR *p* < 0.05) across the preterm period. Right: PPI networks derived using STRING. Top functional enrichments of molecular pathways are shown where applicable. Genes associated with listed enriched pathway and genes differentially expressed in an animal model of preterm brain injury are highlighted. See https://github.com/garedaba/baby-brains/tree/master/data/gene_lists. FDR, false discovery rate; PPI, protein–protein interaction; STRING, Search Tool for the Retrieval of Interacting Genes/Proteins.

**Fig 7:**
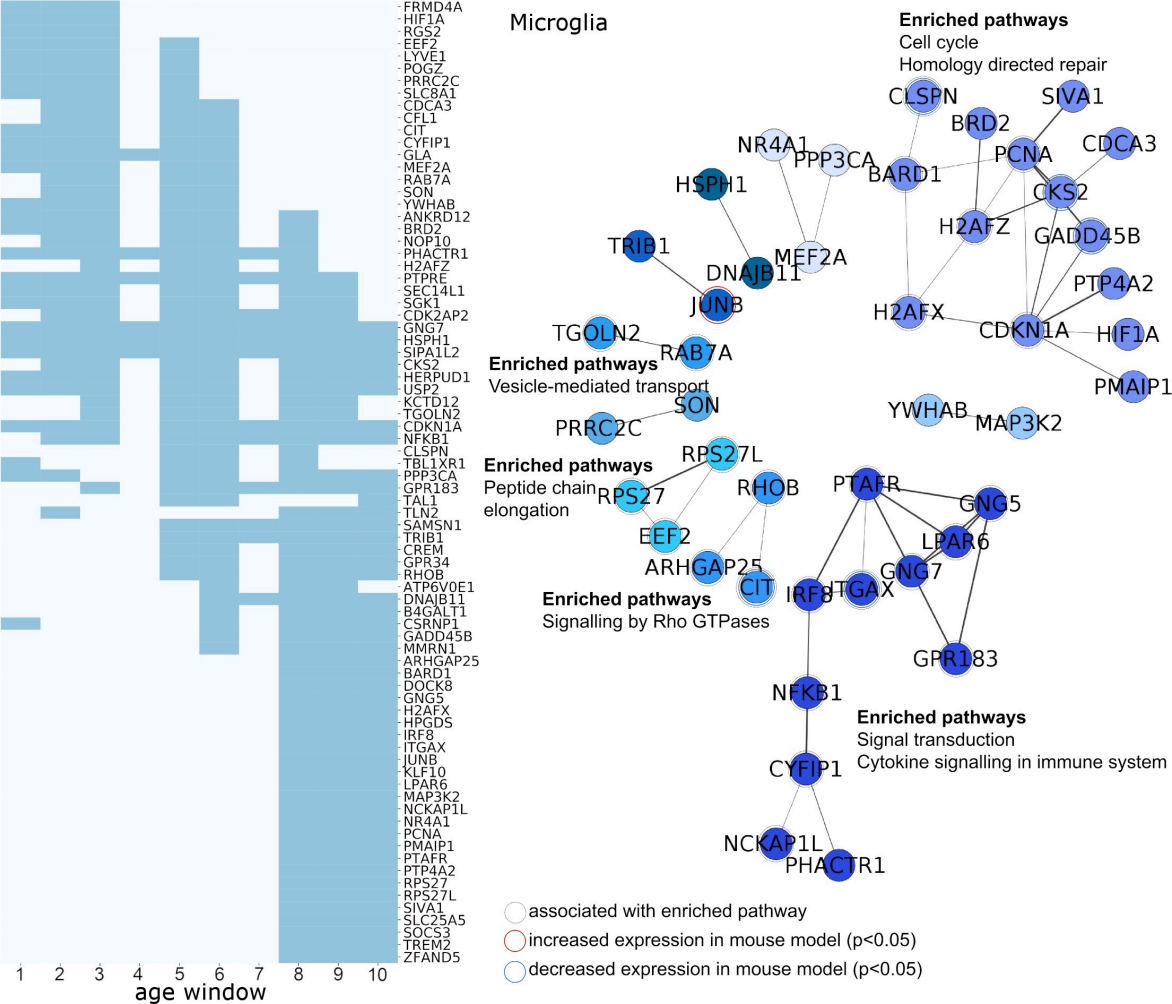
Cellular pathways associated with genes expressed by microglia and developmental alterations in the preterm cortex. Left: Genes expressed by microglia in the fetal cortex and significantly associated with group differences in T1w/T2w contrasts across at least 3 age windows are shown. Dark blue indicates periods where gene expression and T1w/T2w contrast were significantly correlated for each gene (FDR *p* < 0.05) across the preterm period. Right: PPI networks derived using STRING. Top functional enrichments of molecular pathways are shown where applicable. Genes associated with listed enriched pathway and genes differentially expressed in an animal model of preterm brain injury are highlighted. See https://github.com/garedaba/baby-brains/tree/master/data/gene_lists. FDR, false discovery rate; PPI, protein–protein interaction; STRING, Search Tool for the Retrieval of Interacting Genes/Proteins.

To identify potential molecular pathways associated with the neuroimaging differences we observed in the preterm cortex, we identified genes expressed by glial cell types associated with both neuroimaging differences in the human preterm brain and experimental models of preterm brain injury. Using genes expressed by oligodendrocytes (Fig 6A), microglia (Fig 7), and endothelial cells (S11 Fig) and associated with T1/T2 differences across multiple prenatal age windows, we identified protein–protein interaction (PPI) networks using the Search Tool for the Retrieval of Interacting Genes/Proteins (STRING) database [58]. Networks for oligodendrocytes and microglia are shown in Figs 6 and 7. We performed a functional enrichment analysis of Reactome pathways [59] using the whole genome as a reference to identify specific molecular processes involving genes in each PPI network and identified significantly enriched pathways in each cell population (S10–S12 Tables).

In oligodendrocytes, pathway enrichment analysis revealed significant gene associations across multiple time windows. Genes involved in N-methyl-D-aspartate (NMDA) signalling in the mitogen-activated protein kinase (MAPK)/extracellular signal-regulated kinase (ERK) pathway (HSA-438066, HSA-442729, and HSA-442982; *DLG1* and *GRIN2A*) were significantly correlated with T1w/T2w differences across the majority of the preterm period. In contrast, regional expression of genes associated with the MyD88 and Toll-like receptor (TLR) signalling cascades (HSA-975871; *S100B* and *RPS6KA2*) were most closely correlated with T1w/T2w differences in the latter stages of gestation (windows 5 to 9 and 7 to 10, respectively). Other pathways linked genes expression over multiple time periods. Neurotrophin signalling pathways included genes *OMG* (correlated between windows 1 to 8) and *ARHGEF10* (windows 2 to 5), and the Rho-GTPase signalling pathway (HSA-194840) included both *ARHGEF10* and *RHOB* (windows 5 to 10). Finally, sphingolipid metabolism pathways included genes expressed across both the full prenatal in humans and differentially expressed after fetal ischaemic insult in mice (*ACER3*, windows 2 to 9).

In microglia, enriched pathways among genes associated with T1/T2 differences in the preterm cortex included signal transduction (HSA-162582), cytokine signalling (HSA-1280215), and stress response (Homology directed repair; HSA-5693538). The regional expression of genes associated with Rho GTPase signalling (HSA-194315; *CIT*, *RHOB*, and *ARHGAP25*) spanned the prenatal period and were linked to brain injury in the mouse model (*CIT*). Similarly, *ITGAX*, associated with T1/T2 differences late in gestation and linked to cellular inflammatory responses, was differentially expressed in the mouse brain after fetal hypoxic–ischaemia.

In endothelial cells, a large PPI network enriched for genes associated with apoptotic mechanisms, including *CASP3*, *CDKN1A*, and *GADD45B*, each with patterns of expression correlated with T1/T2 differences in the preterm cortex (S11 Fig).

This highlights metabolic signalling pathways associated with genes expressed in developmental glial populations during the period most at risk of interruption by preterm birth with a regional specificity correlated with neuroimaging markers of preterm brain injury at birth and a functional role in experimental models of preterm brain injury.

## Discussion

In this study, we aimed to test the hypothesis that noninvasive neuroimaging measures of cortical structure at birth encode differential spatiotemporal patterning of genes underlying corticogenesis. We found that gene expression in the fetal cortex is mirrored by a principal mode of variation across multiple MRI metrics in the neonatal cortex. Specifically, regional variation in cortical morphometry and microstructure reflects differences in developmental maturity and tissue composition across cortical areas, indexed by the differential timing of gene expression across multiple cell types in the fetal cortex. Having established this relationship, we found that interruption to gestation through preterm birth resulted in significant disruptions to MRI-based measures of cortical development by the time of full-term birth. Further, the effects of preterm birth are temporally and spatially coincident to developmental processes involving cortical glial cell populations. This work provides an experimental framework to link molecular developmental mechanisms to macroscopic measures of cortical anatomy in early life, demonstrating the relationship between fetal gene expression and neonatal brain development and highlighting the specific impact of early exposure to the extrauterine environment due to preterm birth.

Using advanced MRI acquired close to the time of birth in a large, healthy neonatal population, we mapped multiple measures of regional cortical morphometry onto a single mode of variation, defining a principal axis of the neonatal cortex. Ordering of cortical regions along this axis separated lower-order sensory and motor regions from higher-order regions including parietal, frontal, and superior temporal cortex (STC) situated at opposite ends. The shared spatial ordering of cortical properties is a common organisational feature of the mammalian brain [1,2,60,61], reflected in regional variations in cell populations [61], gene expression [22,62], and connectivity [60] as well as MRI-based measures of functional topography [5] and cortical morphometry in both adults [63] and infants [64]. The optimal mapping of cortical properties onto 1 or 2 lower dimensions remains an area of active research [1]; however, studies have demonstrated that variation along 1 axis, or gradient, is largely reflected by concerted changes in others [2,6,65], suggesting that lower-order representations of cortical organisation largely capture shared views of latent neurobiological variation. An important benefit of this framework is the reduction of multiple metrics into a single measure per participant. In our case, this takes advantage of the inherent redundancy across multiple structural and diffusion MRI measures of the same cortical regions, producing a latent representation of cortical structure across scales. Here, we applied a simple linear mapping, arranging cortical regions along a single axis using PCA. This was sufficient to explain a significant proportion of variation in regional MRI-based metrics, with areas with similar cortical profiles clustering together along the principal axis. Based on an observed differential expression of genes associated with specific progenitor cell populations, order along this axis was correlated with spatial gene expression patterns that reflected a differential timing of cortical development across regions. Comparison to cell-specific gene expression profiles in late gestation suggested that MRI-based measures of cortical structure at birth correlated with gene expression by specific glial populations involving oligodendrocytes, microglia, and endothelial cells. This correlation potentially reflects a spatial variation in the developmental timing of processes associated with myelination, neuronal guidance, and the continued maturation of the brain’s vascular networks at around the time of birth [66–70].

The advent of modern transcriptomic technologies has enabled detailed analyses of the foundational molecular mechanisms underpinning corticogenesis in the human fetal brain [16,52,53]. Resolved to the level of individual cells, recent studies have performed systematic explorations of gene expression dynamics across cell cycle progression, migration, and differentiation of several major cell types in the fetal brain [51]. Combined with regional expression levels of bulk tissue mRNA measured across multiple cortical areas, this allows the spatiotemporal mapping of cell-specific gene expression profiles in the developing brain [52]. Here, we used a development atlas of gene expression, measured across 11 cortical regions from 12 to 37 pcw in 16 separate brain specimens [18]. This data resource provides unparalleled access to both the spatial and temporal dynamics of developmental mechanisms ongoing in the cortex during gestation. We found that a number of genes vary across cortical areas in line with a principal imaging axis. In particular, we found that genes with relatively higher expression in higher-order regions during gestation were associated with developmentally earlier processes including neuronal differentiation and migration and were predominantly expressed by intermediate precursor cells and early-maturing inhibitory neurons. Using an alternative approach in 4 mid-gestation brain samples (aged 16 to 21 pcw), Miller and colleagues identified a generally rostro–caudal gradient of gene expression progressing along the contours of the developing brain and anchored in frontal and temporal cortex [16]. While some overlap was evident, 72/85 (85%) of frontally enriched genes that were included in both studies were also positively correlated with the imaging axis; this indicates that variation along PC1 may reflect a combination of multiple overlapping intrinsic hierarchies or cellular gradients underlying cortical development [18,61,62]. Using a machine learning approach designed to accommodate the large number of genes assayed and validated in an independent sample, we established that the maturation of a given tissue sample could be accurately determined based on temporally evolving profiles of gene expression. This approach takes advantage of the degree of variation in gene expression over development. Temporal variability in expression is present across most protein-coding genes, and over 95% of genes that are differentially expressed across cortical regions are also differentially expressed across gestation, with age explaining a large proportion of variance in gene expression [18,19]. Using the relative advancement or delay in predicted age across regional tissue samples, we observed a correlation between emerging differences in areal gene expression and cortical structure at birth, suggesting an interaction between the relative rate of development across regions and length of gestation. This was most notable in the protracted developmental trajectory of the visual cortex in mid-gestation, as noted elsewhere [16]. Overall, our results lend evidential support to the presence of heterogeneous corticogenic timing over gestation [71,72].

Based on these observations, we hypothesised that interruption to gestation would lead to cortical disruptions along the principal cortical axis, reflecting a deleterious interaction with genetically determined developmental programs ongoing in the cortex in the latter stages of gestation. To test this, we compared cortical development in healthy newborns to a cohort of preterm-born infants scanned at the time of normal birth. In line with previous observations [28,43,73], we found significant differences across most cortical metrics of macro- and microstructure in the preterm brain. The magnitude of differences between cohorts aligned with the principal imaging axis, suggesting a differential impact of perinatal adversity on cortical development that is potentially encoded by a selective vulnerability across regions due to differential maturation rates. Adverse intrauterine environments can result in altered patterns of fetal gene expression and brain development [74–79], and we demonstrate overlapping genetic associations between alterations in preterm cortical structure and differential expression in an experimental model of fetal hypoxic–ischaemic brain injury [57]. However, the antecedents and impacts of preterm birth on brain development are multifactorial, and we remain cautious on speculating about the causal mechanisms that may underlie the relationships observed in this study without further empirical evidence.

The largest effect was observed in the myelin-sensitive T1w/T2w contrast. In adults, regional variation in cortical T1w/T2w contrast is highly correlated with quantitative MRI measures of intracortical myelin and histological maps of cytoarchitecture [80]. Myelination in the neonatal cortex is minimal; however, T1w and T2w signal varies as a function of position in the neonatal cortex, and the transcortical pattern of T1w/T2w ratio observed in this study mirrors closely with that reported in older cohorts, with high values predominant in primary sensory regions [80]. In addition, we find that genes with expression correlated with T1w/T2w contrast are enriched for genes expressed by glial cells, including microglia and oligodendrocytes, across the second half of gestation. This mirrors earlier reports, based on microarray data, of correlations between neonatal imaging phenotypes and glial gene expression during gestation [81]. Using a time-resolved analysis, we found several molecular pathways involving genes with spatial and temporal correlation to the potential timing of preterm birth. This method leveraged nonlinear models fit using the full prenatal sample, allowing the discrete mapping of varying gene expression associations across the mid to late fetal period. We identify co-expressed networks of genes expressed by microglia and oligodendrocytes in late gestation and associated with Rho-GTPase signalling pathways, critical for neuronal migration [82], and involved in oligodendrocyte maturation and myelination [83,84]; cytokine signalling and inflammatory response pathways involving NF-κB1 and associated with microglial activation after hypoxic insult [85]; the MAPK/ERK signalling pathway, associated with neuronal and oligodendrocyte proliferation [86,87], as well as sphingolipid metabolic pathways and apoptotic pathways expressed in endothelial cells. These data provide supporting evidence to the important role of developmental glial populations in preterm brain injury [88–90]. We have previously identified risk alleles in preterm-born infants in genes involved in lipid metabolism and microglial activation in the developing brain and associated with altered patterns of brain development by term-equivalent age [91,92]. In this study, we validate our observations in an experimental model of fetal hypoxic–ischaemic injury highlighting a differential expression of glial-expressed genes after early brain injury [57]. We found several genes identified in both human and mouse studies and associated with T1/T2 differences in the preterm brain that were differentially expressed after early brain injury suggested potential deleterious effects on glial cell populations that could lead to disrupted neuronal migration and formation of neural circuitry in the preterm brain [57,57,93–96]. While we recognise the need for further experimental research to elucidate the link to alterations in cortical structure, our findings highlight potential pathways by which preterm birth can result in altered cortical development due to coincidental timing with corticogenic processes in the fetal cortex.

We note several limitations to our study. While we have taken care to validate our observations across several datasets, we are careful to avoid causal language to describe the associations presented. Further experimental evidence is required to fully understand how spatial gene expression gradients lead to alterations in MRI-based measures of cortical structure in healthy and preterm brains. Due to the nature of the data, we compare postmortem gene expression data from the fetal cortex with in vivo measurements of cortical development in healthy and preterm neonates at the end of gestation. We recognise that this approach depends upon a number of assumptions including that gene expression patterns can be generalised across preterm and healthy cohorts and that MRI-based metrics acquired at a single time point act as a surrogate measure for ongoing associations between cortical structure and gene expression during gestation. We mitigate some of these risks by performing validation experiments in independent datasets and comparing our findings to experimental models of preterm brain injury. To measure contemporaneous associations in imaging and gene expression, other studies [50] have employed postmortem MRI of fetal brains to acquire data in age-matched samples, but this approach comes with the additional challenges of imaging postmortem tissue. With advancements in fetal MRI, we anticipate that future research will focus on examining imaging–transcriptomic associations across corresponding time points through mid to late gestation using healthy fetal MRI and preterm infants scanned shortly after birth to better capture the temporal evolution of the reported associations.

While the fetal dataset made available through PsychENCODE represents an unprecedented window into spatiotemporal gene expression in the developing brain, the relatively coarse spatial sampling limits our ability to map fine-grained spatial variation or boundaries between primary and secondary areas. Our analyses only begins at 12 pcw, after early gene expression gradients have begun to impose areal differentiation on the developing brain [9]. Similarly, the bulk tissue samples analysed contained fetal transient structures including the marginal zone and subplate, which differ from the cortex in terms of spatial and temporal development [97]. Although we verified our findings in the cortical plate using microarrays from layer-specific dissection in two 21-pcw donor brains, this analysis was limited to a single time point. We anticipate that with the increasing availability of spatially resolved gene expression datasets, advances in fetal and neonatal imaging, as well as layer-specific imaging analysis [98], further exploration of this area will yield interesting insight into early cortical development. In addition, sexual dimorphism in the transient fetal structures of the brain has been reported [99]. While we felt that the relatively small sample size precluded a direct assessment of sex differences in gene expression, we included sex as a factor in all models of gene expression over time and in our analyses of cortical MRI measures.

To examine cell-specific gene expression, we performed a stratification of bulk tissue RNA using gene lists collated from several independent scRNA studies. This method assumes that areal differences in gene expression are due to differences in developmental timing represented by cellular differentiation as well as changes in the proportion of cell types in composite tissue. While we did not test directly whether the same cell types also show areal differences in gene expression, this was previously explored by Fan and colleagues [51]. In 22- to 23-pcw samples, they found that the dominant mode of variation across cells was cell type rather than regional location and that most areal differences were driven by significant variations in tissue composition, as well as differing patterns of maturation. The advent of high-resolution single-cell RNA maps [53] will hopefully lead to future studies where we can more directly test regional development of specific cell types, rather than broader cell classes.

Finally, in this study, we focused on cortical structure rather than function. Future research may explore spatial associations between fetal gene expression and brain function as measured by MRI. In adults, a number of recent analyses have compared MRI metrics to patterns of gene expression reporting significant associations between correlated gene expression and both structural and functional measures [25,100–102]. As discussed above, concerted variation in cortical properties is a common feature across MRI modalities [103,104], and spatial gene associations may be difficult to disentangle across correlated metrics. However, cortical measures that correlate at a single time point may not develop in tandem, and future exploration of temporal development of cortical structure and function in relation to gene expression will yield interesting future research directions in this area.

In conclusion, we show that noninvasive imaging of the cortical structure in the neonatal brain is sensitive to differential spatiotemporal patterns of gene expression during gestation. In addition, we find that disruption to this developmental programming by preterm birth is associated with significant cortical alterations that appear to reflect the selective vulnerability of developing glial populations in the developing cortex.

## Materials and methods

### Ethics statement

The study was approved by the United Kingdom Health Research Authority (Research Ethics Committee reference number: 14/LO/1169) and performed in accordance with the Declaration of Helsinki. Written informed parental consent was obtained for all participants.

### Participants

Infants were recruited and imaged at the Evelina Newborn Imaging Centre, St Thomas’ Hospital, London, UK for the Developing Human Connectome Project (dHCP). Neuroimaging and basic demographic data from the dHCP are available to download from http://www.developingconnectome.org/second-data-release/.

In total, 442 healthy, term-born infants (gestational age at birth >37 weeks) scanned between February 2015 and November 2018 as part of the dHCP were included in this study. From this cohort, *n* = 362 were successfully processed via the dHCP structural processing pipeline (see “Image processing” section below) and included after quality control. Of these, diffusion data from *n* = 296 was successfully processed using both DTI and NODDI pipelines (see “Image processing” section below). A further 4 participants were excluded following a final visual inspection due to cropped anatomical images. Of 107 preterm infants (gestational age at birth <37 weeks) scanned at term-equivalent age during the same period, 1 was excluded due to incomplete demographic data, *n* = 84 completed structural MRI processing, and *n* = 67 passed diffusion processing after quality control. A further *n* = 3 were removed after final visual inspection.

After quality control and image processing, the final cohort comprised *n* = 292 healthy term-born infants (54% male, mean [SD] postmenstrual age at birth = 39.96 [1.10] weeks, mean [SD] age at imaging = 40.94 [1.56] weeks) and *n* = 64 preterm infants scanned at term-equivalent age (59% male; born 32.00 [3.88] weeks and imaged at 40.57 [2.25] weeks).

### Magnetic resonance imaging

MRI was performed on a 3T Philips Achieva (Philips, the Netherlands) using a dedicated neonatal imaging system including a neonatal 32 channel phased array head coil [29]. Infants were imaged without sedation. T1- and T2-weighted anatomical images were acquired alongside diffusion and resting state functional MRI (total acquisition time: 63 minutes).

Inversion recovery T1- and T2-weighted images were acquired in sagittal and axial orientations (in-plane resolution: 0.8 × 0.8 mm^2^, slice thickness: 1.6 mm with 0.8-mm overlap) with TR = 4,795 ms; TI = 1,740 ms; TE = 8.7 ms; Sensitivity Encoding (SENSE): 2.27 (axial) and 2.66 (sagittal) for T1-weighted images and TR = 12,000 ms, TE = 156 ms; SENSE: 2.11 (axial) and 2.60 (sagittal) for T2-weighted images. T1- and T2-weighted image stacks were motion corrected and reconstructed using the multi-slice aligned sensitivity encoding method with integration into a 3D volume using a super-resolution scheme into 0.8 × 0.8 × 0.8 mm resolution volumes [105,106].

Diffusion MRI was acquired with a spherically optimised set of directions over 4 b-shells (b = 0 s/mm^2^: 20 directions; b = 400: 64 directions; b = 1,000: 88 directions; b = 2,600: 128 directions) with a multiband factor acceleration of 4, TR = 3,800 ms; TE = 90 ms; SENSE: 1.2 and acquired resolution of 1.5 mm × 1.5 mm with 3-mm slices (1.5-mm overlap) reconstructed using an extended SENSE technique into 1.5 × 1.5 × 1.5 mm volumes [107,108].

### Image processing

T1- and T2-weighted images were processing using the dHCP structural pipeline (https://github.com/BioMedIA/dhcp-structural-pipeline) [28]. Briefly, T2-weighted images were bias corrected (N4) [109], brain-extracted (Brain Extraction Tool [BET]) [110], and segmented into grey matter, white matter, and cerebrospinal fluid using Developing Brain Region Annotation With Expectation-Maximization (DRAW-EM) [111]. Cortical surfaces of the right and left hemisphere were then extracted [112] and aligned to a population-specific cortical template [113] using spherical inflation and multimodal surface matching (MSM) with higher-order constraints (https://github.com/ecr05/MSM_HOCR) [114,115]. This method ensures that all surfaces across participants have one-to-one vertex correspondence with the dHCP neonatal template. For each participant, we extracted the following metrics: cortical thickness (corrected for cortical curvature) and T1w/T2w contrast (calculated using rigidly aligned T1-weighted images).

Diffusion-weighted images were preprocessed by first denoising [116] and removing Gibbs ringing artefacts [117], followed by a slice-to-volume motion and distortion correction with a slice-level outlier rejection using a multi-shell spherical harmonic signal representation (spherical harmonics and radial decomposition [SHARD]) [118]. Visual inspection of the 4D images ensured motion correction and outlier rejection was successful and that images of poor quality were excluded from further analysis.

We fit each participant’s diffusion data with both a diffusion tensor model, fitted to the b = 1,000 s^2^/mm shell and implemented in MRtrix [119], and the neurite orientation dispersion and density imaging (NODDI) model [120]. For the diffusion data, NODDI was implemented with the NODDI MATLAB toolbox using the “invivopreterm” tissue type options with the default parameters of intrinsic diffusivity fixed to 1.7 × 10^−3^ mm^2^/s and the starting point for values considered as the fraction of intra-neurite space lowered to 0 to 0.3 (instead of 0 to 1 in the adult brain) to better fit higher water content in the newborn compared to the mature adult brain [49,121].

From these models, we derived parametric maps of FA and MD from DTI, as well as maps of ODI, quantifying the angular variation of neurite orientation within a voxel and fICVF indexing the tissue volume fraction restricted within neurites. Cortical diffusion maps were projected to the cortical surface after co-registration with the corresponding anatomical data.

Images were visually inspected after acquisition and after reconstruction and following each processing pipeline. Any images that failed to successfully complete the processing pipelines or failed visual inspection at any stage were removed from further analysis. As an additional step, we quantified in-scanner movement and image quality using a summary metric of the total head translation, rotation, and the ratio of detected outlier slices. These 3 metrics were combined into 1 aggregated quality assurance measure [118,122]. This measure did not significantly differ between groups (term [mean +/− SD] = 1.61 +/− 1.79, preterm = 1.55 +/− 1.03; t = 0.24, *p* = 0.81). Including QA as a covariate in our analyses of group differences did not impact our reported observations.

### Bulk tissue gene expression data

Preprocessed, bulk tissue cortical gene expression data were made available as part of the PsychENCODE project (available to download from http://development.psychencode.org/) [18]. Tissue was collected after obtaining parental or next of kin consent and with approval by the institutional review boards at the Yale University School of Medicine, the National Institutes of Health, and at each institution from which tissue specimens were obtained.

Tissue processing is detailed elsewhere [18]. In brief, mRNA data were available for postmortem human brain tissue collected from *n* = 41 specimens aged between 8 pcw and 40 postnatal years. For each brain, regional dissection of up to 16 cerebral regions was performed, including 11 neocortical regions (DLPFC, VLPFC, orbitofrontal cortex [OFC], medial frontal cortex [MFC], M1, S1, IPC, A1C, STC, ITC, and V1) and 5 subcortical regions (hippocampus, amygdala, striatum, thalamus, and cerebellar cortex). Detailed anatomical boundaries for each cortical region at each stage of development are provided elsewhere [17,18]. Regional tissue samples were subject to mRNA-seq using an Illumina Genome Analyzer IIx (Illumina, San Diego, California, United States of America) and mRNA-seq data processed using RSEQtools (version 0.5) [123]. Gene expression was measured as RPKM. Conditional quantile normalisation was performed to remove GC content bias and ComBat used to remove technical variance due to processing site (Yale or University of Southern California) [18,124,125].

In this study, we included RPKM data from neocortical samples of prenatal specimens aged 12 pcw onwards (*n* = 16, age range = 12 to 37 pcw, mean [SD] age = 18.4 [7.7] pcw, 50% male, mean [SD] number of cortical regions sampled = 9.75 [1.6], mean [SD] postmortem interval = 7.1 [12.6] hours, mean [SD] RNA integrity number [RIN] [126] = 9.26 [0.73]). Prenatal specimens from the earliest developmental window (8 to 9 pcw) were excluded as some cortical regions (e.g., M1 and S1) were combined together to account for immature cortical anatomy [17,18].

The prenatal gene expression data were initially filtered to only include protein-coding genes (NCBI GRCh38.p12, *n* = 18,524 out of a possible 20,720). In order to restrict our analysis to focus on genes expressed in the developing cortex, we further filtered this list to only contain genes expressed by cells in the fetal cortex based on the composite list of prenatal cell markers from 5 independent single-cell RNA studies of the developing fetal cortex (see “Genetic markers of cell type” section below). This resulted in expression data from a final set of 5,287 genes.

### Additional gene expression datasets

#### BrainCloud

Preprocessed microarray data from *n* = 46 human prefrontal cortex tissue samples aged approximately 95 to 390 postconceptional days (14 pcw to 4 months postnatal age) were downloaded from the Gene Expression Omnibus (GEO; accession: GSE30272). Prior to analysis, individual gene expression was modelled using nonlinear splines. Surrogate variable analysis was performed to remove technical variation and batch effects (31 surrogate variables) while retaining variation due to age. For further details, please see Colantuoni and colleagues [56]. For each gene, expression was Z-transformed prior to modelling. In total, gene expression for 4,986/5,287 fetal gene markers was available.

#### Single-cell RNA

Regional single-cell RNA gene expression data was made available via GEO (accession: GSE103723) [51]. Briefly, 4,213 single cells were isolated from 20 anatomical regions of the 22- and 23-pcw fetal cortex and subject to single-cell RNA-seq. Normalised expression data in transcripts per million (TPM) were available for 96 cells per tissue sample. We selected data from single cells extracted from matching cortical regions to those described above and classified to 1 of 10 classes based on clusters identified in [51], as detailed in the “Genetic markers of cell type” section below (*n* = 572 cells).

#### Laser microdissection (LMD) microarray data

LMD microarray data were accessed via the BrainSpan data portal (brainspan.org/lcm). This provides access to DNA microarray data from 4 mid-gestational brains, dissected into around 300 anatomical samples. Detailed information is provided elsewhere [16]. We performed a differential search to identify microarray probes with differential expression in the cortical plate of the DLPFC (regional identification: fCPdl), VLPFC (fCPvl), and IPC (pCPpv) compared to M1 (fCPm1) and S1 (fCPs1) measured in two 21-pcw donor brains. Differential expression data for 23,000 probes were downloaded and corresponding data mapped to the set of preselected fetal marker genes for comparison.

#### Experimental mouse model of preterm brain injury

Gene expression levels in P1.5 mouse cortex were measured for control or ischaemic pups, where ischaemia was induced by maternal uterine artery occlusion at E16.5. Four mice in each group were included [57]. Data were made available via GEO (accession: GSE89998). Analysis was performed using “GEO2R” and the “limma” package (www.ncbi.nlm.nih.gov/geo/geo2r). Expression data were first log2-transformed before fold-change was estimated across groups. Mouse genes were mapped to human homologs using Ensembl (www.ensemble.org) and matched to the list of human fetal gene markers.

### Cortical regions of interest

To facilitate comparison between developmental RNA and MRI data, we created a set of cortical ROI labels corresponding to the anatomical dissections used for mRNA analysis and aligned to the dHCP imaging data.

Reference postmortem MRI data were acquired as part of the Allen Institute BrainSpan Atlas of the Developing Human Brain. Details of tissue processing and MRI acquisition are available at https://help.brain-map.org/download/attachments/3506181/BrainSpan_MR_DW_DT_Imaging.pdf. In brief, MRI was acquired at 3T (Siemens, Germany) in a postmortem, whole-brain specimens aged 22 pcw. In addition, anatomical annotations corresponding to the regional dissections in Miller and colleagues [16], Kang and colleagues [17], and Li and colleagues [18] were provided on a reconstructed cortical surface from a 19-pcw prenatal specimen [50]. Cortical ROI data were available to download in VTK file format, separately for left and right cortical hemispheres (S1 Fig).

To generate a set of dHCP-compatible cortical labels, we reconstructed the cortical surface of a 3T postmortem MRI image from a 22-pcw brain. First, manually creating a brain mask to remove non-brain tissue, then smoothing using a mean filter of 3-mm width. We performed automated tissue segmentation on the smoothed image using the dHCP structural pipeline, manually correcting tissue segmentations on a slice-by-slice basis for accuracy prior to cortical surface reconstruction. Using dHCP tools, the fetal cortical surface was extracted and cortical labels manually transferred onto it based on the reference labels provided by Huang and colleagues [50] and anatomical descriptions in Li and colleagues [18]. Finally, the fetal surface was inflated to a sphere and co-registered to the earliest time point (36 weeks gestational age) of the dHCP cortical surface atlas using MSM [113,115].

This resulted in a set of 11 cortical ROI, each associated with regional bulk tissue mRNA data sampled across gestation and co-registered with dHCP neuroimaging data to allow correspondent sampling of cortical imaging metrics in the neonatal brain (S1 Fig).

### Cortical imaging metric analysis

For every participant, mean values of each imaging metric (thickness, T1w/T2w contrast, FA, MD, fICVF, and ODI) were calculated within each cortical ROI. Metric values were averaged across hemispheres and outlier values identified and removed using a median absolute deviation (MAD) of >3.5.

For all healthy term-born infants, regional metrics were Z-transformed and averaged across participants to produce a group average region ✕ metric matrix representing the relative variation of each imaging metric across cortical regions.

We projected the group average data onto 2 axes using PCA via eigendecomposition of the data covariance matrix. This results in a set of *L* eigenvectors, *W*_*L*_, which map the original *n*×*p* data matrix, *X* onto a set of orthogonal axes as *T*_*L*_ = *XW*_*L*_. As generally, *L*<*p*, the truncated *n*×*L* matrix, *T*_*L*_, forms a low-dimensional representation of the original data. We can then project each participant’s region ✕ metric matrix, *X*_*s*_, onto a common set of axes as $T_{L}^{s}=X_{s}W_{L}$, where $T_{L}^{s}$ represents the *L* component scores for each participant, *s*.

All analysis was performed in Python (3.7.3) using Scipy (1.3.0) [127] and Scikit-Learn (0.21.2) [128].

### Modelling gene expression trajectories

For each gene, we modelled the relationship between gene expression and specimen age using mixed-effects models. Using bulk tissue RPKM data described above, each gene’s expression data were first Winsorised to set very small or large outlying values to the fifth and 95th centile values, respectively, to stabilise against extreme values before log2 transformation.

We compared 2 models, modelling regional gene expression as either a linear or nonlinear function of age with fixed effects of sex and RIN. We accounted for sample-specific variation by including in the model a random intercept for each specimen, such that

y∼f(v)+Xβ+Zb,

where *f*(·) is a nonlinear function of predictor *v*, *X* is an *m*-observation ×*p* design matrix modelling *p* linear, fixed effects, and *Z* is an *m*×(*n*·*r*) design matrix modelling *r* random effects across *n* specimens. In this case, age was included as either a nonlinear predictor, *f*(*v*), or as a fixed linear effect alongside sex and RIN. We specified a relatively smooth nonlinear function of age using a natural cubic spline with 4 knots evenly spaced across the age span. To estimate region-specific trajectories, we calculated a second nonlinear model, additionally including separate smooth functions for each cortical region. Models were compared using AIC and BIC.

We calculated age-corrected RPKM values for each gene in all cortical samples using the residuals of the best-fit nonlinear mixed model (S3 Fig) to test the spatial association between gene expression and the principal imaging gradient using nonparametric correlation (Kendall’s *τ*).

Modelling was performed in R (3.6.1) using “nlme” [129] and “mgcv” [130] packages.

### Genetic markers of cell type

Genetic markers of cortical cell types were collated from 5 independent single-cell RNA studies of the fetal cortex [18,51–54]. Using single-cell RNA-seq, each study identified sets of genes differentially expressed across cell clusters or types. Cell types were independently defined in each study, and a list of all cell types included in this study (*n* = 87) are shown in S3 Table. Where applicable, for a given cell type, DEGs were included as cell type markers if they were found to be expressed in at least 50% all cells surveyed [18,51,52]. Across all 5 studies, each cell type was manually assigned to 1 of 11 cell classes based on text descriptions from each study (astrocyte, endothelial cell, microglia, neuron:excitatory, neuron:inhibitory, neuron:unclassified, oligodendrocyte, oligodendrocyte precursor cell [OPC], pericyte, intermediate progenitor cell, and radial glia) and classified as either a precursor or mature cell type (S3 Table). For each cell class, omnibus gene lists were created by collating identified gene markers for all cell types within a class. Unique gene lists were created by excluding any genes identified as a marker of more than 1 cell class.

### Cell type embedding

Using the region-specific, nonlinear model specified above, expression trajectories for every gene were estimated for each region at 50 evenly spaced points across the full observation window (12 pcw to 37 pcw). For each cell type identified in the fetal cortex (see above), expression trajectories for all cell type gene markers were normalised to unit length, concatenated over regions, and averaged to capture both temporal and spatial variation in average gene expression across cell types. Similarities between cell type gene expression trajectories were then visualised by embedding into a 2D space using UMAP based on Euclidean distance.[131]

### Enrichment analyses

We performed ORA of each list of gene markers for each of 10 cell classes (excluding neuron:unclassified), calculating the hypergeometric statistic

p=1−∑i=0x(Ki)(M−KN−i)(MN),

where *p* is the probability of finding *x* or more genes from a cell class–specific gene list *K* in a set of randomly selected genes, *N* drawn from a background set, *M*. We calculated enrichment ratios as the proportion of cell class–specific genes in the gene list of interest, compared to the proportion in the full background set. The background gene set was defined as the full list of protein-coding genes included in the analysis (*n* = 5,287) unless otherwise specified. We corrected for multiple comparisons across cell classes using FDR.

We additionally performed ORA for GO terms using WebGestalt [55].

### Weighted gene correlation network analysis

We used WGCNA [132] to identify co-expression modules within PC+ and PC− gene sets. We performed topology analysis using a gene × gene adjacency matrix constructed from the residualised log2-transformed RPKM data, after accounting for variance due to age, sex, and sample effects (see “Modelling gene expression trajectories” section above). A soft threshold was chosen to approximate scale free topology in the adjacency matrix (PC+: power = 5, r^2^ = 0.77; PC−: power = 10, r^2^ = 0.78) [133] before transformation into a topological overlap matrix. Hierarchical clustering was used to assign genes to modules based on the dynamic tree-cutting method [134]. Analysis was performed in R (3.6.1) with the “WGCNA” package [132].

### Predicting tissue maturity

We used gene expression over time to construct a predictive model of genetic maturity using SVR. To maximise coverage across the prenatal period, we included additional samples aged 8 pcw up to 4 months of postnatal age (*n* = 21 total). Using the *n* = 120 regionally varying genes (PC+ and PC−), we first calculated regional gene expression profiles, corrected for variance due to sex, RIN, and specimen identification while retaining variance due to age, using nonlinear mixed-effects models. We then averaged gene expression across cortical regions in each specimen to create a specimen × gene (21 × 120) mean gene expression matrix, where each row represents the normalised log2(RPKM) of each gene for a given specimen, averaged across cortical regions.

In machine learning, kernels can be applied high-dimensional datasets to improve model fitting where *n*<<*p*. To calculate regional variation in genetic maturity, we implemented a leave-one-out (LOO) model using SVR with a linear kernel (Scikit-Learn; regularisation parameter set to *C* = 10.0) and modelling the association between specimen age (in postconceptional days) and mean cortical gene expression data in 20 out of 21 specimens. We then used this model to predict age using the regional gene expression profiles of the remaining, left-out specimen, resulting in 11 age predictions, 1 per cortical region. We repeated this process, leaving out a different specimen each time.

In order to estimate a stable prediction of tissue maturity, we repeated the modelling using a bootstrapped selection of genes, repeating gene sampling with replacement 1,000 times. We also repeated the modelling using all 5,287 genes. We calculated the correlation between regional genetic maturity (averaged over 1,000 bootstraps) and PC1 score for each specimen and tested the significance of this relationship by permuting mean gene expression profiles with respect to specimen age 5,000 times during model training.

### Group comparison of cortical morphology

We compared regional cortical metrics in term and preterm cohorts using a linear mixed effects modelling approach. For each of 6 metrics, we modelled metric value as a combination of age, sex, regional PC1 score and birth group status (term or preterm). We included an interaction term for PC1 and birth status to test the hypothesis that preterm birth incurs differential effects across cortical regions in line with PC1. We also included participant identification as a random effect to account for correlated within-participant observations across regions. We fit nested models by maximum likelihood, comparing model fits with and without the inclusion of birth status using AIC and BIC (S6 Table).

### Developmental gene enrichment

In order to test cell class enrichment over time, we split the preterm period (approximately 160 to 260 postconceptional days) into 10 age windows. Using nonlinear gene expression trajectories, calculated across cortical regions (see “Modelling gene expression trajectories” section above), we averaged modelled gene expression within each window for every cortical region. Then, in each window, we calculated the nonparametric association (Kendall’s *τ*) between gene expression and the mean difference between term and preterm groups in T1w/T2w contrast in each cortical region and recorded significantly associated genes (FDR-corrected at *p* < 0.05). Finally, we performed cell-class enrichment (see “Enrichment analyses” section above) in each of the 10 time-resolved gene sets.

### PPI networks

PPI networks were visualised in Cytoscape (3.7.2) using the StringDB protein query. Pathway enrichment of Reactome pathways was performed for subnetworks.

## Supporting information

S1 Fig Creating matching cortical labels to enable spatial comparisons of transcriptomic and neuroimaging data. Eleven cortical ROI corresponding to the anatomical tissue samples used to measure prenatal gene expression (left) were transferred onto a postmortem fetal cortical surface reconstruction (middle) to allow registration to the dHCP neonatal surface atlas (right) and sampling of neuroimaging metrics from anatomical correspondent regions at term-equivalent age. A1, primary auditory cortex; DLPFC, dorsolateral prefrontal cortex; IPC, inferior parietal cortex; ITC, inferior temporal cortex; M1, primary motor cortex; MFC, medial frontal cortex; OFC, orbitofrontal cortex; S1, primary sensory cortex; STC, superior temporal cortex; V1, primary visual cortex; VLPFC, ventrolateral prefrontal cortex.
(TIF)

S2 Fig Contribution of each cortical metric to the principal imaging component. Left: The principal eigenvector for PC1 shows the contribution of each metric to the principal component. Right: correlations between group average regional cortical metrics and PC1. See https://github.com/garedaba/baby-brains/tree/master/figures.
(TIF)

S3 Fig Calculating spatial correlations between gene expression and imaging metrics. For each gene, expression (RPKM) over samples was modelled using nonlinear mixed effects models, accounting for variation due to age, sex, and specimen. The residuals of this model, representing age-corrected expression levels in each cortical region for every sample, were correlated with the regional principal component score yielding a nonparametric association (Kendall’s τ) and *p*-value. Genes were ranked based on association with the imaging phenotype and *p*-values corrected with FDR to select significantly associated gene sets. See https://github.com/garedaba/baby-brains/tree/master/figures.
(PNG)

S4 Fig Gene modules negatively associated with PC1. Gene co-expression analysis of all negatively associated (PC−) genes revealed 3 modules (1N, 2N, and 3N). Intra-modular connections are shown with node size and colour indicating strength and edge thickness and colour indicating weight. See https://github.com/garedaba/baby-brains/tree/master/results/wgcna.
(TIF)

S5 Fig Cell class embeddings coloured by different features. UMAP embedding of 86 cell types based on trajectories of relative gene expression coloured by study, timing, and neuronal subtype. See https://github.com/garedaba/baby-brains/tree/master/figures.
(TIF)

S6 Fig Cell-specific variation of PC+ and PC− genes. Using single-cell RNA-seq data, we calculated total RNA expression (sum of cell TPM) of all genes in the PC+ and PC− gene sets. Individual cells were clustered based on cell assignments into each of 10 cell classes. No cells were annotated to radial glia, oligodendrocyte, or pericyte in these regions. See https://github.com/garedaba/baby-brains/tree/master/figures.
(TIF)

S7 Fig Developmental trajectories of genetic maturation for regions. Regions are grouped and ordered by position along PC1 (left to right: A1C, S1, M1; MFC, STC; V1; DLPFC, VLPFC, OFC, IPC, ITC). Density plots show the difference between model-predicted age and sample age (5,000 bootstrapped gene samples). Positions to the left of 0 indicate regions that are less mature compared to the mean. Five time windows through gestation are shown, with age in postconceptional days. See https://github.com/garedaba/baby-brains/tree/master/figures.
(TIF)

S8 Fig Tissue maturity correlates with regional variation in cortical morphometry at birth using all fetal marker genes. Left: the relationship between predicted and true sample age for all regional samples (*n* = 198 samples from *n* = 21 brains) in the PsychENCODE dataset aged between 50 and 400 postconceptional days (8 pcw to 4 postnatal months), estimated using SVR and LOO cross-validation. The SVR model was validated using additional samples from the BrainCloud dataset (*n* = 46 samples). Shaded area indicates 95% CI. Right. Correlation between PC1 score and predicted age is shown for each sample. Error bars show 95% CI for regional age predictions (1,000 bootstrapped gene samples). See https://github.com/garedaba/baby-brains/tree/master/figures.
(TIF)

S9 Fig Cortical metric data from all participants projected into PC space. Individual data were projected onto the first 2 principal components calculated from the group average data matrix. Points are coloured by PC1 score, and black outline indicates data from preterm infants. See https://github.com/garedaba/baby-brains/tree/master/figures.
(TIF)

S10 Fig Mean group differences in cortical metrics. Scatterplots show the average group difference (term − preterm) in regional metrics as a function of PC1 score. Linear regressions with 95% CI are shown. See https://github.com/garedaba/baby-brains/tree/master/figures.
(TIF)

S11 Fig Cellular pathways associated with genes expressed by endothelial cells and developmental alterations in the preterm cortex. Left: Genes expressed by endothelial cells in the fetal cortex and significantly associated with group differences in T1w/T2w contrasts across at least 3 age windows are shown. Dark red indicates periods where gene expression and T1w/T2w contrast were significantly correlated for each gene (FDR *p* < 0.05) across the preterm period. Right: protein–protein interaction networks derived using STRING. Top functional enrichments of molecular pathways are shown where applicable. Genes associated with listed enriched pathway and genes differentially expressed in an animal model of preterm brain injury are highlighted. See https://github.com/garedaba/baby-brains/tree/master/data/gene_lists.
(TIF)

S1 Table Gene Ontology enrichment of genes positively associated genes with PC1 using different background reference sets. (DOCX)

S2 Table Gene Ontology enrichment of top 100 genes differentially expressed in LMD dataset using different background reference sets. (DOCX)

S3 Table Cell type classification by class and timing. (DOCX)

S4 Table Cell class enrichments for PC1 including all genes within class. (DOCX)

S5 Table Cell class enrichments for PC1 including only unique genes within class. (DOCX)

S6 Table Model fits for each cortical metric with and without inclusion of birth status. (DOCX)

S7 Table Model parameter estimates for each cortical metric. (DOCX)

S8 Table Estimated marginal means of each cortical metric for preterm and term cohorts. (DOCX)

S9 Table Cell class enrichments for differentially expressed genes in mouse model of fetal hypoxia. (DOCX)

S10 Table Enriched pathways within PPI networks in oligodendrocytes. (DOCX)

S11 Table Enriched pathways within PPI networks in microglia. (DOCX)

S12 Table Enriched pathways within PPI networks in endothelial cells. (DOCX)

## Abbreviations

A1C: primary auditory cortex
AIC: Akaike information criterion
BET: Brain Extraction Tool
BIC: Bayesian information criterion
DEG: differentially expressed gene
dHCP: Developing Human Connectome Project
DLPFC: dorsolateral prefrontal cortex
DRAW-EM: Developing Brain Region Annotation With Expectation-Maximization
DTI: diffusion tensor imaging
ERK: extracellular signal-regulated kinase
FA: fractional anisotropy
FDR: false discovery rate
fICVF: intracellular volume fraction
GEO: Gene Expression Omnibus
GO: Gene Ontology
IPC: inferior parietal cortex
ITC: inferior temporal cortex
LMD: laser microdissection
LOO: leave-one-out
M1: primary motor cortex
MAD: median absolute deviation
MAPK: mitogen-activated protein kinase
MD: mean diffusivity
MFC: medial frontal cortex
MRI: magnetic resonance imaging
mRNA-seq: mRNA sequencing
MSM: multimodal surface matching
NMDA: N-methyl-D-aspartate
NODDI: neurite orientation dispersion and density imaging
ODI: orientation dispersion index
OFC: orbitofrontal cortex
OPC: oligodendrocyte precursor cell
ORA: overrepresentation analysis
PCA: principal component analysis
pcw: postconceptional weeks
PPI: protein–protein interaction
RIN: RNA integrity number
RNA-seq: RNA sequencing
ROI: regions of interest
RPKM: reads per kilobase of transcript per million mapped reads
S1: primary sensory cortex
SENSE: sensitivity encoding
SHARD: spherical harmonics and radial decomposition
STC: superior temporal cortex
STRING: Search Tool for the Retrieval of Interacting Genes/Proteins
SVR: support vector regression
TPM: transcripts per million
TLR: Toll-like receptor
UMAP: Uniform Manifold Approximation and Projection
V1: primary visual cortex
VLPFC: ventrolateral prefrontal cortex
WGCNA: weighted gene correlation network analysis
