# Supplementary figures and images for: Cortical morphology at birth reflects spatiotemporal patterns of gene expression in the fetal human brain
Source: PLoS Biol. 2020 Nov 23;18(11):e3000976. doi: 10.1371/journal.pbio.3000976 (PMC7721147; doi:10.1371/journal.pbio.3000976)

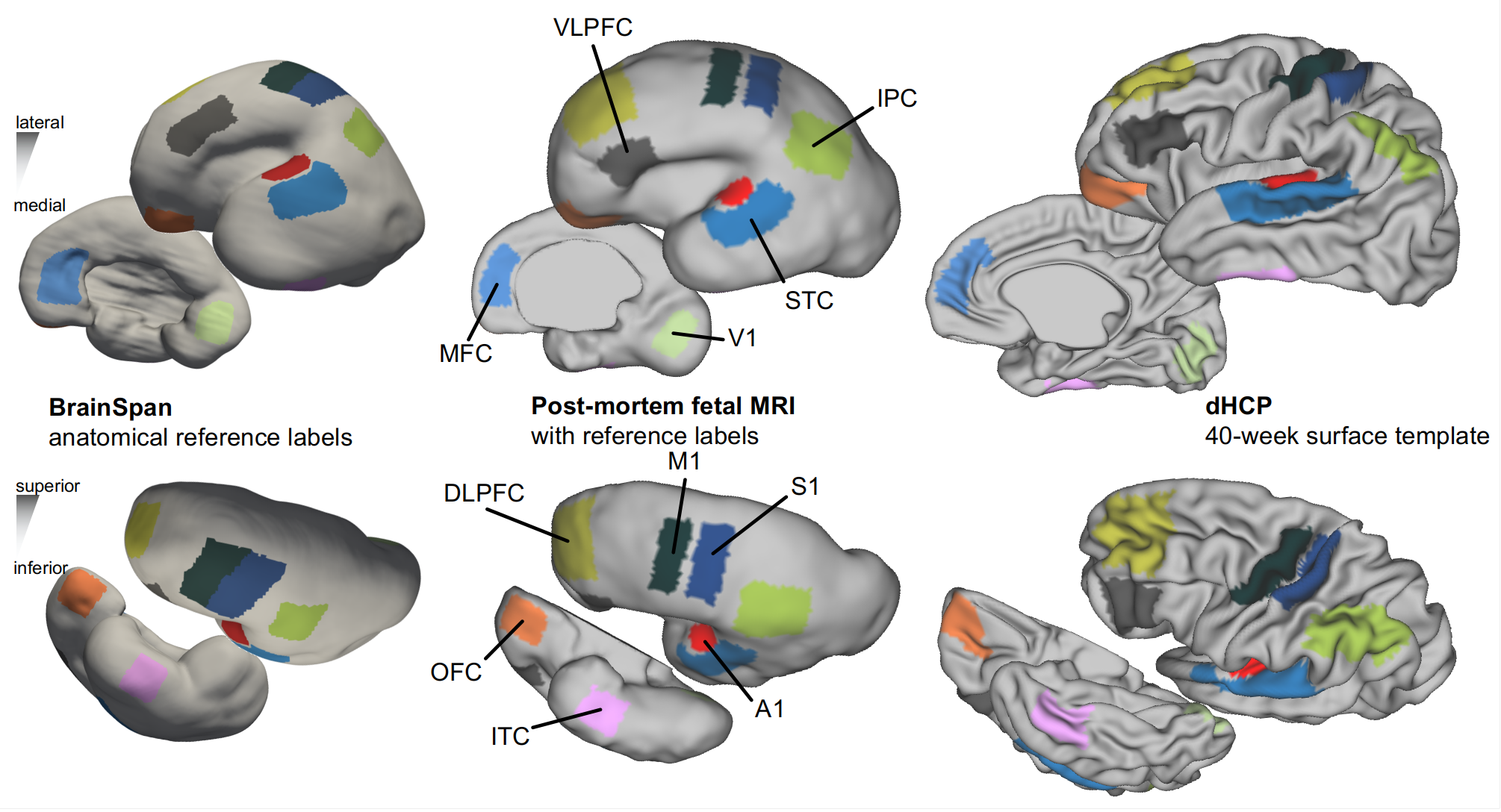

Supplement: S1 Fig — Eleven cortical ROI corresponding to the anatomical tissue samples used to measure prenatal gene expression (left) were transferred onto a postmortem fetal cortical surface reconstruction (middle) to allow registration to the dHCP neonatal surface atlas (right) and sampling of neuroimaging metrics from anatomical correspondent regions at term-equivalent age. A1, primary auditory cortex; DLPFC, dorsolateral prefrontal cortex; IPC, inferior parietal cortex; ITC, inferior temporal cortex; M1, primary motor cortex; MFC, medial frontal cortex; OFC, orbitofrontal cortex; S1, primary sensory cortex; STC, superior temporal cortex; V1, primary visual cortex; VLPFC, ventrolateral prefrontal cortex. (TIF) [file pbio.3000976.s001.tif]

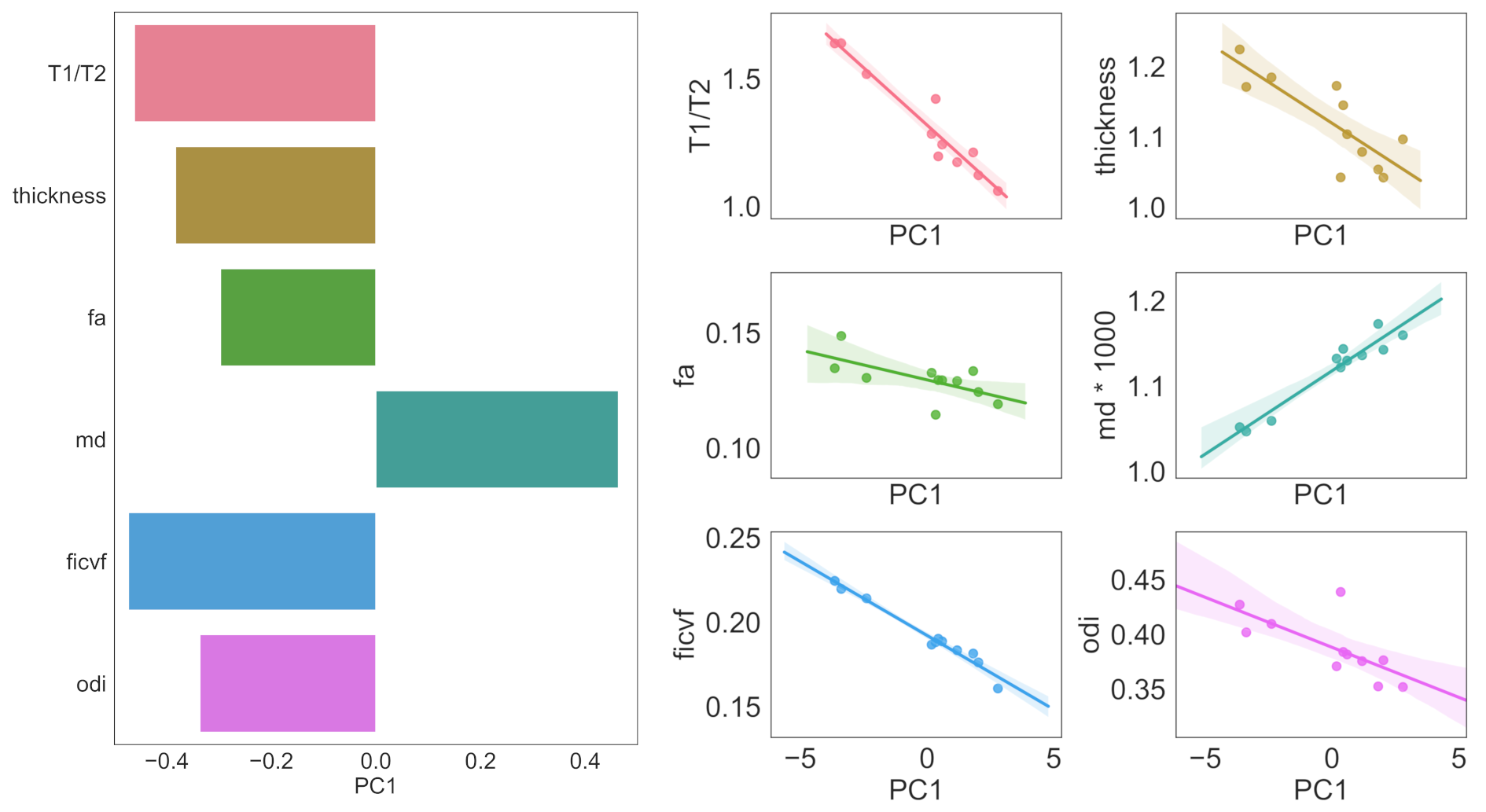

Supplement: S2 Fig — Left: The principal eigenvector for PC1 shows the contribution of each metric to the principal component. Right: correlations between group average regional cortical metrics and PC1. See https://github.com/garedaba/baby-brains/tree/master/figures. (TIF) [file pbio.3000976.s002.tif]

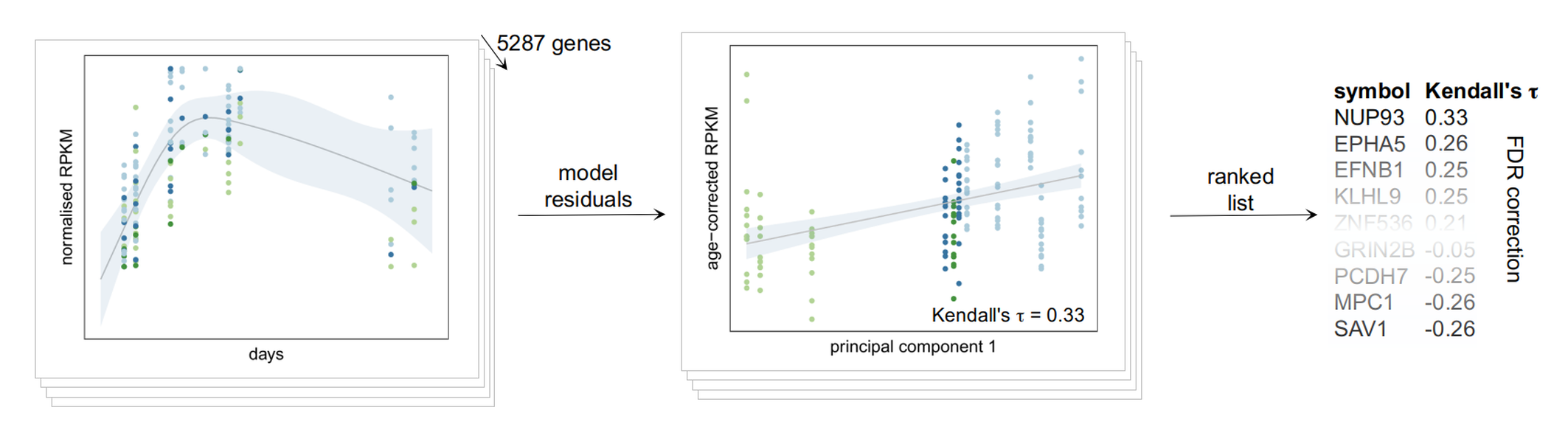

Supplement: S3 Fig — For each gene, expression (RPKM) over samples was modelled using nonlinear mixed effects models, accounting for variation due to age, sex, and specimen. The residuals of this model, representing age-corrected expression levels in each cortical region for every sample, were correlated with the regional principal component score yielding a nonparametric association (Kendall’s τ) and p-value. Genes were ranked based on association with the imaging phenotype and p-values corrected with FDR to select significantly associated gene sets. See https://github.com/garedaba/baby-brains/tree/master/figures. (PNG) [file pbio.3000976.s003.png]

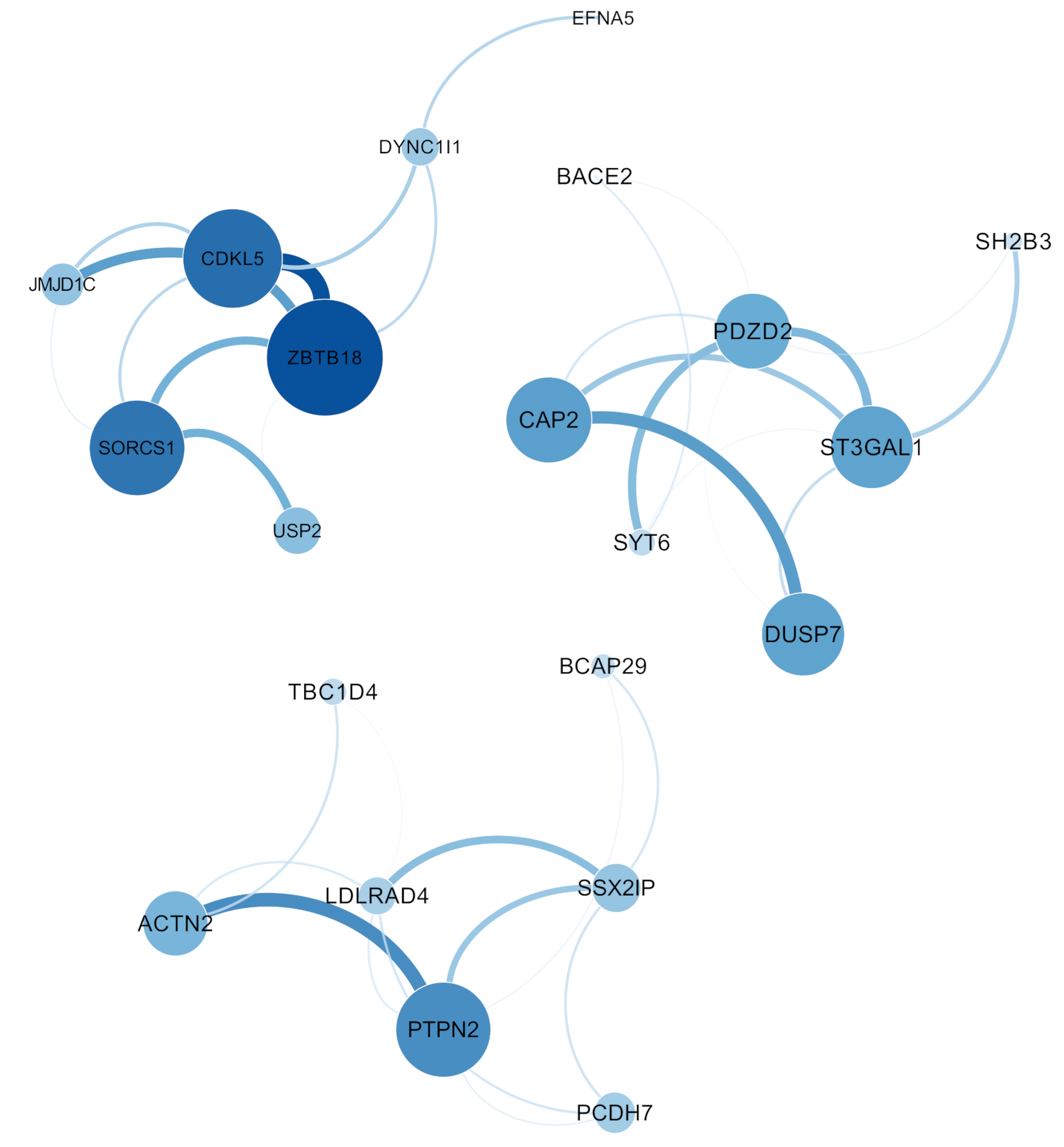

Supplement: S4 Fig — Gene co-expression analysis of all negatively associated (PC−) genes revealed 3 modules (1N, 2N, and 3N). Intra-modular connections are shown with node size and colour indicating strength and edge thickness and colour indicating weight. See https://github.com/garedaba/baby-brains/tree/master/results/wgcna. (TIF) [file pbio.3000976.s004.tif]

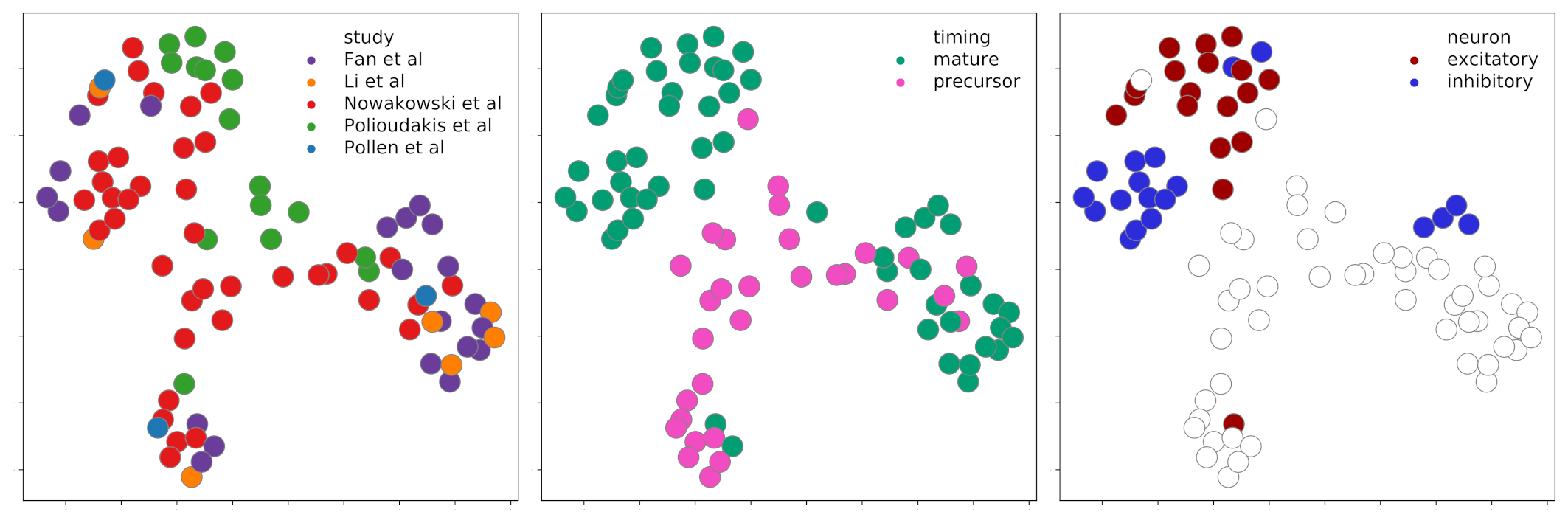

Supplement: S5 Fig — UMAP embedding of 86 cell types based on trajectories of relative gene expression coloured by study, timing, and neuronal subtype. See https://github.com/garedaba/baby-brains/tree/master/figures. (TIF) [file pbio.3000976.s005.tif]

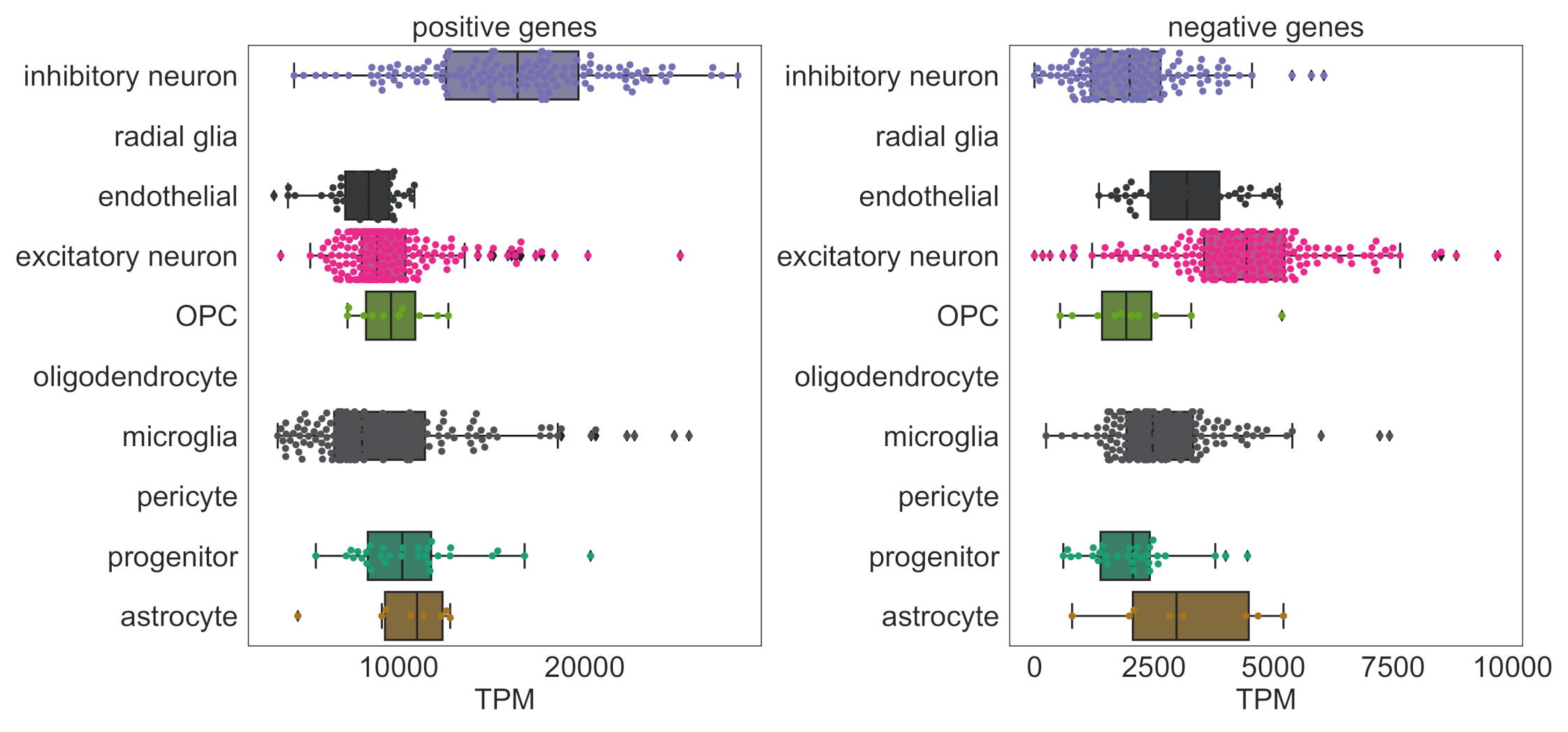

Supplement: S6 Fig — Using single-cell RNA-seq data, we calculated total RNA expression (sum of cell TPM) of all genes in the PC+ and PC− gene sets. Individual cells were clustered based on cell assignments into each of 10 cell classes. No cells were annotated to radial glia, oligodendrocyte, or pericyte in these regions. See https://github.com/garedaba/baby-brains/tree/master/figures. (TIF) [file pbio.3000976.s006.tif]

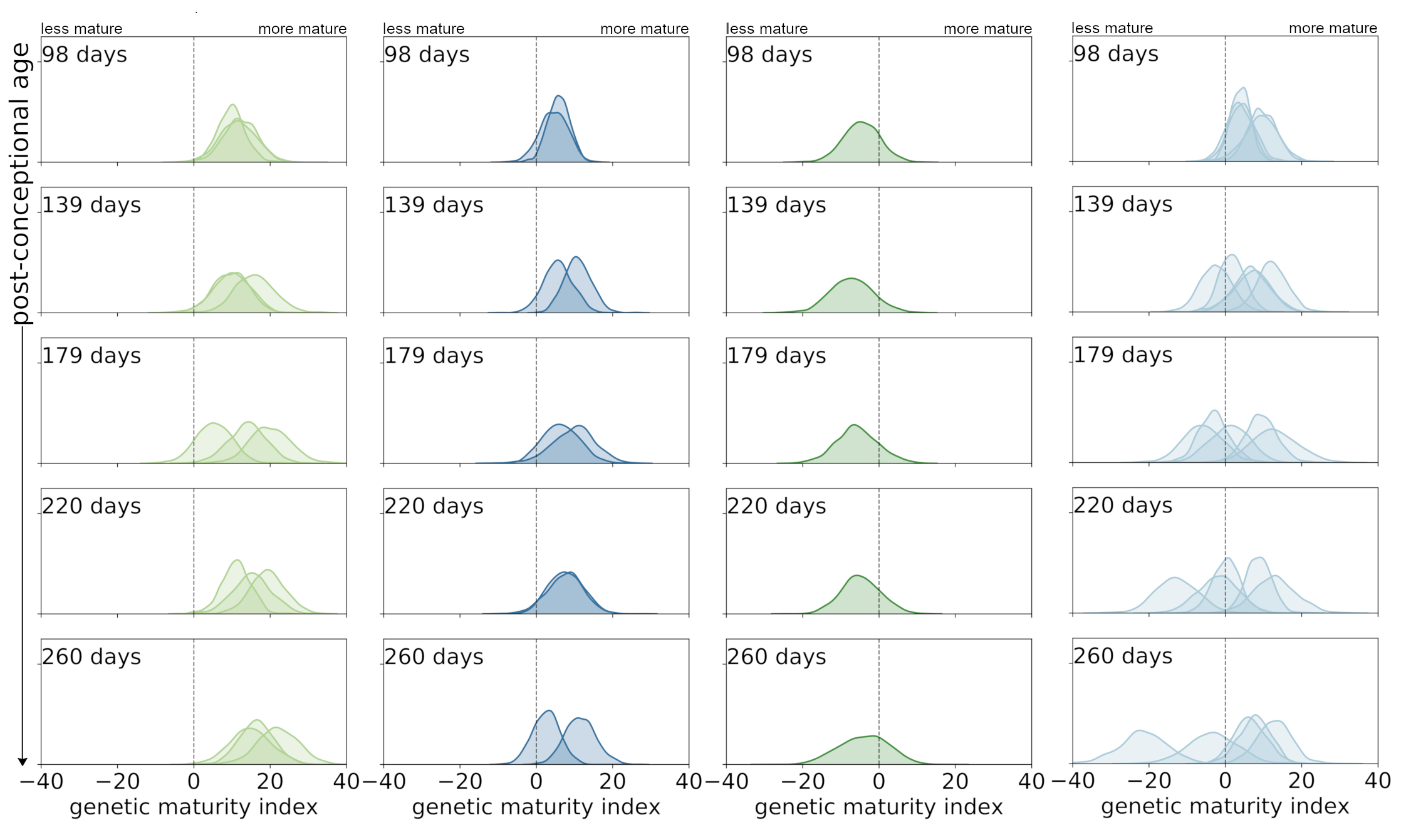

Supplement: S7 Fig — Regions are grouped and ordered by position along PC1 (left to right: A1C, S1, M1; MFC, STC; V1; DLPFC, VLPFC, OFC, IPC, ITC). Density plots show the difference between model-predicted age and sample age (5,000 bootstrapped gene samples). Positions to the left of 0 indicate regions that are less mature compared to the mean. Five time windows through gestation are shown, with age in postconceptional days. See https://github.com/garedaba/baby-brains/tree/master/figures. (TIF) [file pbio.3000976.s007.tif]

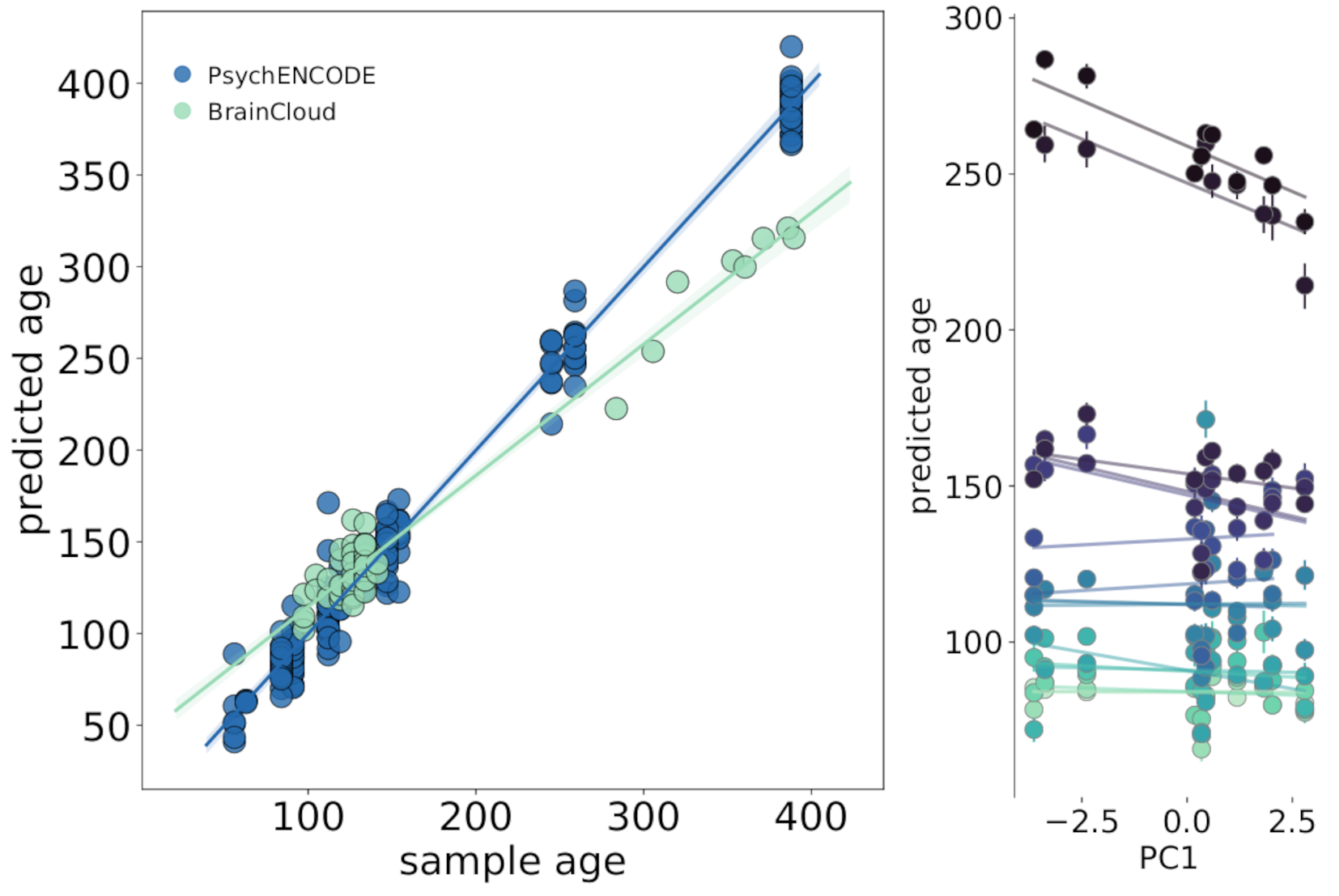

Supplement: S8 Fig — Left: the relationship between predicted and true sample age for all regional samples (n = 198 samples from n = 21 brains) in the PsychENCODE dataset aged between 50 and 400 postconceptional days (8 pcw to 4 postnatal months), estimated using SVR and LOO cross-validation. The SVR model was validated using additional samples from the BrainCloud dataset (n = 46 samples). Shaded area indicates 95% CI. Right. Correlation between PC1 score and predicted age is shown for each sample. Error bars show 95% CI for regional age predictions (1,000 bootstrapped gene samples). See https://github.com/garedaba/baby-brains/tree/master/figures. (TIF) [file pbio.3000976.s008.tif]

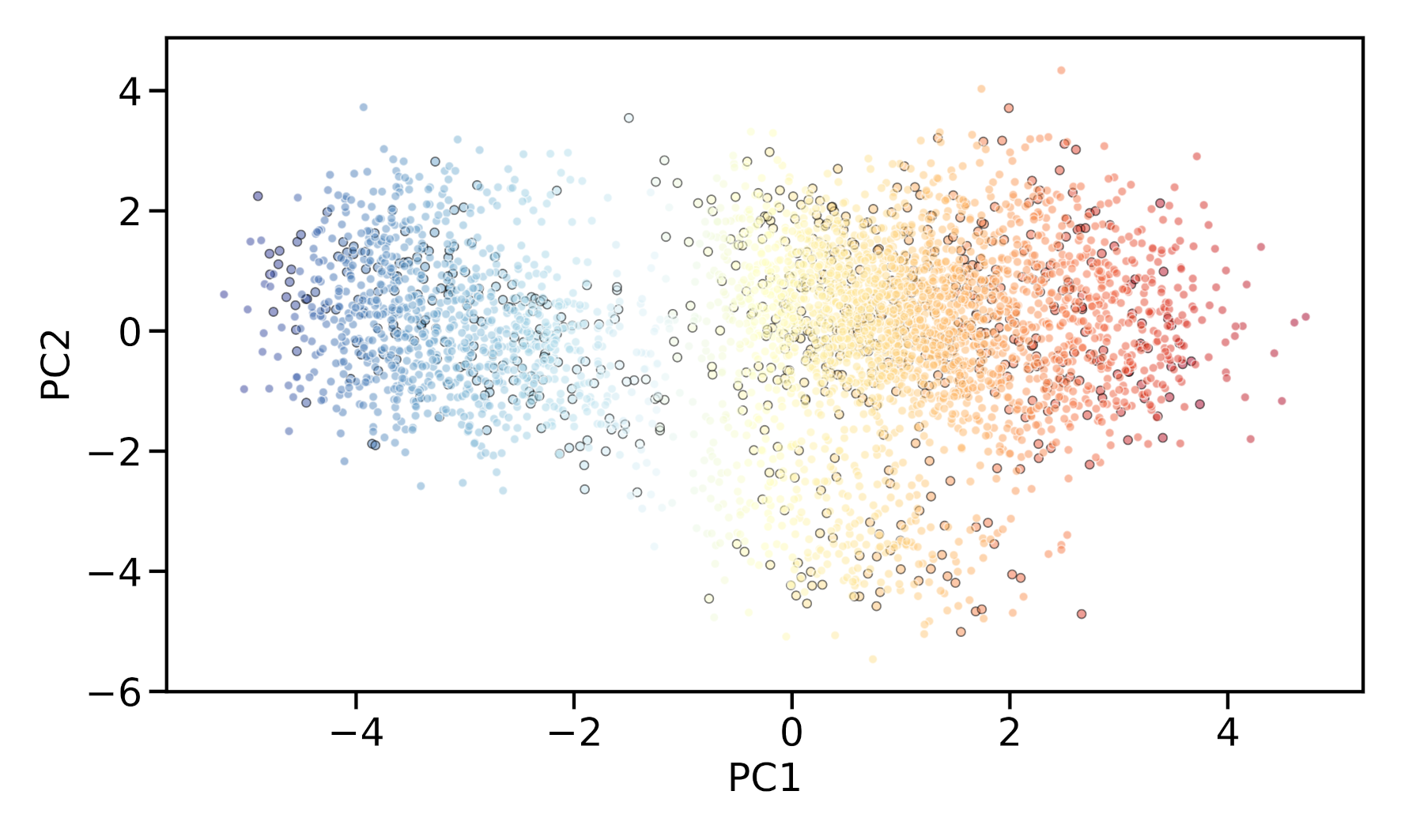

Supplement: S9 Fig — Individual data were projected onto the first 2 principal components calculated from the group average data matrix. Points are coloured by PC1 score, and black outline indicates data from preterm infants. See https://github.com/garedaba/baby-brains/tree/master/figures. (TIF) [file pbio.3000976.s009.tif]

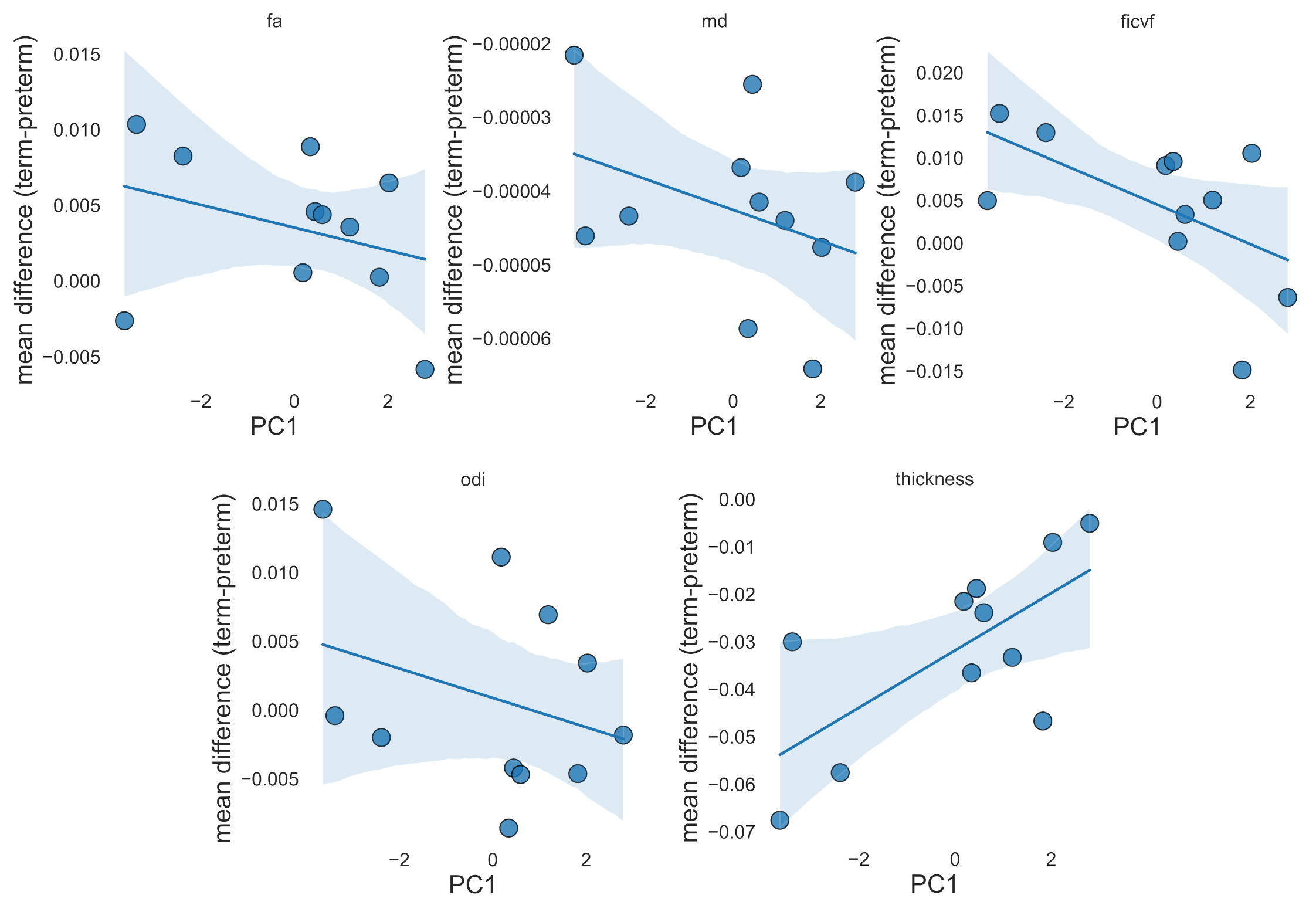

Supplement: S10 Fig — Scatterplots show the average group difference (term − preterm) in regional metrics as a function of PC1 score. Linear regressions with 95% CI are shown. See https://github.com/garedaba/baby-brains/tree/master/figures. (TIF) [file pbio.3000976.s010.tif]

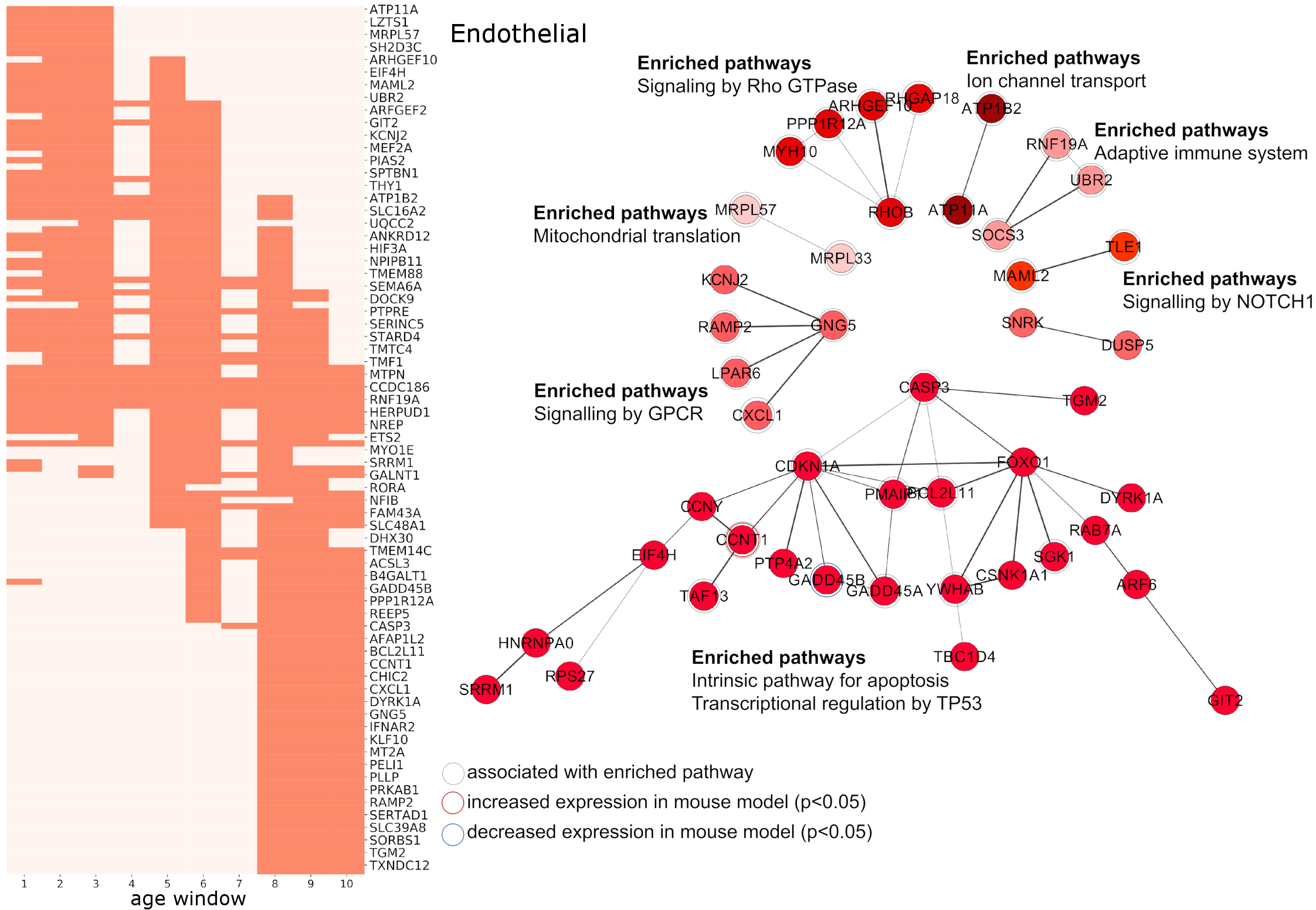

Supplement: S11 Fig — Left: Genes expressed by endothelial cells in the fetal cortex and significantly associated with group differences in T1w/T2w contrasts across at least 3 age windows are shown. Dark red indicates periods where gene expression and T1w/T2w contrast were significantly correlated for each gene (FDR p < 0.05) across the preterm period. Right: protein–protein interaction networks derived using STRING. Top functional enrichments of molecular pathways are shown where applicable. Genes associated with listed enriched pathway and genes differentially expressed in an animal model of preterm brain injury are highlighted. See https://github.com/garedaba/baby-brains/tree/master/data/gene_lists. (TIF) [file pbio.3000976.s011.tif]
