# Supplementary material for: Cortical morphology at birth reflects spatiotemporal patterns of gene expression in the fetal human brain
Source: PLoS Biol. 2020 Nov 23;18(11):e3000976. doi: 10.1371/journal.pbio.3000976 (PMC7721147; doi:10.1371/journal.pbio.3000976)
Supplement: S1 Table — (DOCX) [file pbio.3000976.s012.docx]

**S1 Table: Gene Ontology enrichment of genes positively associated genes with PC1 using different background reference sets**

|  |  | **Reference set** | | | |
| --- | --- | --- | --- | --- | --- |
|  |  | **All genes (n=18,524)** | | **Fetal gene markers (n=5287)** | |
| **GO term**^†^ | **Description** | **Enrichment** | **FDR** | **Enrichment** | **FDR** |
| GO:0048863 | stem cell differentiation | 9.33 | 0.0013* | 6.38 | 0.0059* |
| GO:0021953 | central nervous system neuron differentiation | 9.23 | 0.0022* | 5.90 | 0.0161* |
| GO:0045165 | cell fate commitment | 7.48 | 0.0022* | 6.52 | 0.0059* |
| GO:0030900 | forebrain development | 5.66 | 0.0042* | 3.57 | 0.0646 |
| GO:0007389 | pattern specification process | 4.90 | 0.0102* | 4.91 | 0.0098* |
| GO:0010721 | negative regulation of cell development | 5.29 | 0.0354* | 3.28 | 0.2513 |
| GO:0001764 | neuron migration | 7.94 | 0.0367* | 4.80 | 0.2135 |
| GO:0060485 | mesenchyme development | 5.35 | 0.0653 | 4.06 | 0.2135 |
| GO:0033002 | muscle cell proliferation | 6.48 | 0.0653 | 4.36 | 0.2513 |
| GO:0001667 | ameboidal-type cell migration | 4.38 | 0.0653 | 2.75 | 0.3308 |

*p<0.05 after correction for multiple comparisons

^†^ top 10 terms with ‘all genes’ background set are listed
