# Supplementary material for: Cortical morphology at birth reflects spatiotemporal patterns of gene expression in the fetal human brain
Source: PLoS Biol. 2020 Nov 23;18(11):e3000976. doi: 10.1371/journal.pbio.3000976 (PMC7721147; doi:10.1371/journal.pbio.3000976)
Supplement: S2 Table — (DOCX) [file pbio.3000976.s013.docx]

**S2 Table: Gene Ontology enrichment of top 100 genes differentially expressed in LMD dataset using different background reference sets**

|  |  | **Reference set** | | | |
| --- | --- | --- | --- | --- | --- |
|  |  | **All genes (n=18,524)** | | **Fetal gene markers (n=5287)** | |
| **GO term**^†^ | **Description** | **Enrichment** | **FDR** | **Enrichment** | **FDR** |
| GO:0021953 | central nervous system neuron differentiation | 6.86 | 0.0198* | 4.66 | 0.1053 |
| GO:0097581 | lamellipodium organisation | 10.92 | 0.0198* | - | - |
| GO:0051493 | regulation of cytoskeleton organisation | 3.93 | 0.0198* | 2.25 | 0.4328 |
| GO:0007626 | locomotory behaviour | 6.26 | 0.0198* | 4.58 | 0.1053 |
| GO:0032970 | regulation of actin-filament-based process | 3.94 | 0.1180 | 2.76 | 0.4328 |
| GO:0007015 | actin-filament organisation | 3.72 | 0.1414 | 2.59 | 0.4502 |
| GO:0002009 | morphogenesis of an epithelium | 3.27 | 0.1414 | 2.71 | 0.4328 |
| GO:0061564 | axon development | 3.23 | 0.1414 | 1.89 | 0.6246 |
| GO:0048638 | regulation of developmental growth | 3.91 | 0.1451 | 2.78 | 0.4502 |
| GO:0031532 | actin cytoskeleton reorganisation | 7.19 | 0.1456 | - | - |

*p<0.05 after correction for multiple comparisons

^†^ top 10 terms with ‘all genes’ background set are listed
