# Supplementary material for: Cortical morphology at birth reflects spatiotemporal patterns of gene expression in the fetal human brain
Source: PLoS Biol. 2020 Nov 23;18(11):e3000976. doi: 10.1371/journal.pbio.3000976 (PMC7721147; doi:10.1371/journal.pbio.3000976)
Supplement: S3 Table — (DOCX) [file pbio.3000976.s014.docx]

**S3 Table: Cell-type classification by class and timing**

| **Cell type description** | **Study** | **Class** | **Timing** |
| --- | --- | --- | --- |
| Astro_1 | Fan | astrocyte | mature |
| Astro_2 | Fan | astrocyte | mature |
| Endo_1 | Fan | endothelial | mature |
| Endo_2 | Fan | endothelial | mature |
| Micro_1 | Fan | microglia | mature |
| Micro_2 | Fan | microglia | mature |
| Micro_3 | Fan | microglia | mature |
| Ex_1 | Fan | neuron:excitatory | mature |
| Ex_2 | Fan | neuron:excitatory | mature |
| Ex_4 | Fan | neuron:excitatory | mature |
| In_1 | Fan | neuron:inhibitory | mature |
| In_2 | Fan | neuron:inhibitory | mature |
| In_3 | Fan | neuron:inhibitory | mature |
| In_4 | Fan | neuron:inhibitory | mature |
| In_5 | Fan | neuron:inhibitory | mature |
| In_6 | Fan | neuron:inhibitory | mature |
| In_7 | Fan | neuron:inhibitory | mature |
| In_8 | Fan | neuron:inhibitory | mature |
| Olig | Fan | oligodendrocyte | mature |
| OPC | Fan | OPC | precursor |
| NSC_1 | Fan | progenitor | precursor |
| NSC_2 | Fan | progenitor | precursor |
| Astro_prenatal | Li | astrocyte | mature |
| Endo_prenatal | Li | endothelial | mature |
| Microglia_prenatal | Li | microglia | mature |
| ExN_prenatal | Li | neuron:excitatory | mature |
| InN_prenatal | Li | neuron:inhibitory | mature |
| Oligo_prenatal | Li | oligodendrocyte | mature |
| NPC_prenatal | Li | progenitor | precursor |
| Astrocyte | Nowakowski | astrocyte | mature |
| Endothelial | Nowakowski | endothelial | mature |
| Microglia | Nowakowski | microglia | mature |
| EN-PFC1 | Nowakowski | neuron:excitatory | mature |
| EN-PFC2 | Nowakowski | neuron:excitatory | mature |
| EN-PFC3 | Nowakowski | neuron:excitatory | mature |
| EN-V1-1 | Nowakowski | neuron:excitatory | mature |
| EN-V1-2 | Nowakowski | neuron:excitatory | mature |
| EN-V1-3 | Nowakowski | neuron:excitatory | mature |
| nEN-early1 | Nowakowski | neuron:excitatory | mature |
| nEN-early2 | Nowakowski | neuron:excitatory | mature |
| nEN-late | Nowakowski | neuron:excitatory | mature |
| IN-CTX-CGE1 | Nowakowski | neuron:inhibitory | mature |
| IN-CTX-CGE2 | Nowakowski | neuron:inhibitory | mature |
| IN-CTX-MGE1 | Nowakowski | neuron:inhibitory | mature |
| IN-CTX-MGE2 | Nowakowski | neuron:inhibitory | mature |
| nIN1 | Nowakowski | neuron:inhibitory | mature |
| nIN2 | Nowakowski | neuron:inhibitory | mature |
| nIN3 | Nowakowski | neuron:inhibitory | mature |
| nIN4 | Nowakowski | neuron:inhibitory | mature |
| nIN5 | Nowakowski | neuron:inhibitory | mature |
| OPC | Nowakowski | OPC | precursor |
| IPC-div1 | Nowakowski | progenitor | precursor |
| IPC-div2 | Nowakowski | progenitor | precursor |
| IPC-nEN1 | Nowakowski | progenitor | precursor |
| IPC-nEN2 | Nowakowski | progenitor | precursor |
| IPC-nEN3 | Nowakowski | progenitor | precursor |
| MGE-IPC1 | Nowakowski | progenitor | precursor |
| MGE-IPC2 | Nowakowski | progenitor | precursor |
| MGE-IPC3 | Nowakowski | progenitor | precursor |
| MGE-div | Nowakowski | progenitor | precursor |
| MGE-RG1 | Nowakowski | radial glia | precursor |
| MGE-RG2 | Nowakowski | radial glia | precursor |
| RG-div1 | Nowakowski | radial glia | precursor |
| RG-div2 | Nowakowski | radial glia | precursor |
| RG-early | Nowakowski | radial glia | precursor |
| oRG | Nowakowski | radial glia | precursor |
| tRG | Nowakowski | radial glia | precursor |
| vRG | Nowakowski | radial glia | precursor |
| Endothelial (End) | Polioudakis | endothelial | mature |
| Microglia (Mic) | Polioudakis | microglia | mature |
| Excitatory deep layer 1 (ExDp1) | Polioudakis | neuron:excitatory | mature |
| Excitatory deep layer 2 (ExDp2) | Polioudakis | neuron:excitatory | mature |
| Maturing excitatory (ExM) | Polioudakis | neuron:excitatory | mature |
| Maturing excitatory upper enriched (ExM-U) | Polioudakis | neuron:excitatory | mature |
| Migrating excitatory (ExN) | Polioudakis | neuron:excitatory | mature |
| Interneuron CGE (InCGE) | Polioudakis | neuron:inhibitory | mature |
| Interneuron MGE (InMGE) | Polioudakis | neuron:inhibitory | mature |
| Oligodendrocyte precursor (OPC) | Polioudakis | OPC | precursor |
| Pericyte (Per) | Polioudakis | pericyte | mature |
| Cycling progenitor G2/M phase (PgG2M) | Polioudakis | progenitor | precursor |
| Cycling progenitor S phase (PgS) | Polioudakis | progenitor | precursor |
| IP | Polioudakis | progenitor | precursor |
| oRG | Polioudakis | radial glia | precursor |
| vRG | Polioudakis | radial glia | precursor |
| Neuronal | Pollen | neuron:unclassified† | mature |
| Neural_progenitor | Pollen | progenitor | precursor |
| Radial_glia | Pollen | radial glia | precursor |

† removed from subsequent analysis
