# Supplementary material for: Cortical morphology at birth reflects spatiotemporal patterns of gene expression in the fetal human brain
Source: PLoS Biol. 2020 Nov 23;18(11):e3000976. doi: 10.1371/journal.pbio.3000976 (PMC7721147; doi:10.1371/journal.pbio.3000976)
Supplement: S4 Table — (DOCX) [file pbio.3000976.s015.docx]

**S4 Table: Cell class enrichments for PC1 including all genes within class**

| **PC+ genes** |  |  |  |  |
| --- | --- | --- | --- | --- |
| **class**† | **number genes** | **enrichment ratio** | **p** | **FDR corrected p** |
| astrocyte | 473 | 0.00 | 1.000 | 1.000 |
| endothelial | 1574 | 0.38 | 1.000 | 1.000 |
| microglia | 759 | 0.19 | 1.000 | 1.000 |
| neuron:excitatory | 2186 | 1.09 | 0.300 | 0.857 |
| neuron:inhibitory* | 574 | 3.21 | <0.001 | <0.001 |
| oligodendrocyte | 622 | 0.36 | 0.993 | 1.000 |
| OPC | 496 | 1.05 | 0.504 | 1.000 |
| pericyte | 343 | 0.43 | 0.950 | 1.000 |
| progenitor* | 1750 | 1.63 | <0.001 | 0.003 |
| radial glia | 2416 | 1.21 | 0.073 | 0.367 |
| **PC- genes** |  |  |  |  |
| astrocyte | 473 | 1.14 | 0.449 | 1.000 |
| endothelial | 1574 | 1.31 | 0.111 | 0.445 |
| microglia | 759 | 0.57 | 0.936 | 1.000 |
| neuron:excitatory | 2186 | 1.19 | 0.172 | 0.574 |
| neuron:inhibitory | 574 | 0.19 | 0.997 | 1.000 |
| oligodendrocyte | 622 | 1.75 | 0.056 | 0.367 |
| OPC | 496 | 0.43 | 0.952 | 1.000 |
| pericyte | 343 | 0.31 | 0.963 | 1.000 |
| progenitor | 1750 | 0.24 | 1.000 | 1.000 |
| radial glia | 2416 | 0.40 | 1.000 | 1.000 |

* FDR-corrected p<0.05 †excluding neuron:unclassified
