# Supplementary material for: Cortical morphology at birth reflects spatiotemporal patterns of gene expression in the fetal human brain
Source: PLoS Biol. 2020 Nov 23;18(11):e3000976. doi: 10.1371/journal.pbio.3000976 (PMC7721147; doi:10.1371/journal.pbio.3000976)
Supplement: S5 Table — (DOCX) [file pbio.3000976.s016.docx]

**S5 Table: Cell class enrichments for PC1 including only unique genes within class**

| **PC+ genes** |  |  |  |  |
| --- | --- | --- | --- | --- |
| **class**† | **number genes** | **enrichment ratio** | **p** | **FDR corrected p** |
| astrocyte | 71 | 0.00 | 1.000 | 1.000 |
| endothelial | 568 | 0.39 | 0.986 | 1.000 |
| microglia | 204 | 0.00 | 1.000 | 1.000 |
| neuron:excitatory | 611 | 0.36 | 0.992 | 1.000 |
| neuron:inhibitory* | 25 | 14.00 | <0.001 | 0.006 |
| oligodendrocyte | 334 | 0.44 | 0.945 | 1.000 |
| OPC | 30 | 0.00 | 1.000 | 1.000 |
| pericyte | 3 | 0.00 | 1.000 | 1.000 |
| progenitor | 234 | 2.27 | 0.036 | 0.243 |
| radial glia | 427 | 1.58 | 0.116 | 0.464 |
| **PC- genes** |  |  |  |  |
| astrocyte | 71 | 1.52 | 0.486 | 1.000 |
| endothelial | 568 | 1.72 | 0.074 | 0.370 |
| microglia | 204 | 1.06 | 0.570 | 1.000 |
| neuron:excitatory | 611 | 2.14 | 0.008 | 0.083 |
| neuron:inhibitory | 25 | 0.00 | 1.000 | 1.000 |
| oligodendrocyte | 334 | 1.63 | 0.195 | 0.650 |
| OPC | 30 | 0.00 | 1.000 | 1.000 |
| pericyte | 3 | 0.00 | 1.000 | 1.000 |
| progenitor | 234 | 0.46 | 0.892 | 1.000 |
| radial glia | 427 | 0.25 | 0.984 | 1.000 |

* FDR-corrected p<0.05 †excluding neuron:unclassified
