# Supplementary material for: Cortical morphology at birth reflects spatiotemporal patterns of gene expression in the fetal human brain
Source: PLoS Biol. 2020 Nov 23;18(11):e3000976. doi: 10.1371/journal.pbio.3000976 (PMC7721147; doi:10.1371/journal.pbio.3000976)
Supplement: S6 Table — (DOCX) [file pbio.3000976.s017.docx]

## Table F: Model fits for each cortical metric with and without inclusion of birth status

**S6 Table: Model fits for each cortical metric with and without inclusion of birth status**

|  | **Reduced model*** | | **Full model**† | |  |  |
| --- | --- | --- | --- | --- | --- | --- |
| **Metric** | **AIC** | **BIC** | **AIC** | **BIC** | **AIC_full_-AIC_reduced_** | **BIC_full_-BIC_reduced_** |
| T1/T2 | -7920 | -7883 | -8090 | -8040 | -169.531 | -156.994 |
| thickness | -10840 | -10802 | -10884 | -10834 | -43.884 | -31.343 |
| FA | -23143 | -23105 | -23169 | -23119 | -26.031 | -13.523 |
| MD | -67222 | -67184 | -67281 | -67231 | -59.552 | -47.051 |
| ODI | -15870 | -15832 | -15869 | -15819 | 0.688 | 13.175 |
| fICVF | -22218 | -22180 | -22316 | -22266 | -97.657 | -85.138 |
| *metric ~ 1 + age + male + PC + (1 \| subject) | | | |  |  |  |
| †metric ~ 1 + age + male + PC + term + term:PC + (1 \| subject) | | | |  |  |  |
