# Supplementary material for: Cortical morphology at birth reflects spatiotemporal patterns of gene expression in the fetal human brain
Source: PLoS Biol. 2020 Nov 23;18(11):e3000976. doi: 10.1371/journal.pbio.3000976 (PMC7721147; doi:10.1371/journal.pbio.3000976)
Supplement: S7 Table — (DOCX) [file pbio.3000976.s018.docx]

**S7 Table: Model parameter estimates for each cortical metric**

| **metric** |  | **95% Confidence Interval** | |  |  |
| --- | --- | --- | --- | --- | --- |
| **T1/T2** | **Estimate** | **Lower** | **Upper** | **t** | **p** |
| term - preterm | 1.2E-01 | 9.9E-02 | 1.4E-01 | 1.2E+01 | <0.00001 |
| PC | -8.4E-02 | -8.5E-02 | -8.2E-02 | -1.1E+02 | <0.00001 |
| term:PC | -1.2E-02 | -1.5E-02 | -8.8E-03 | -7.7E+00 | <0.00001 |
| age | 2.7E-02 | 2.2E-02 | 3.1E-02 | 1.1E+01 | <0.00001 |
| male-female | -2.2E-02 | -3.7E-02 | -6.1E-03 | -2.7E+00 | 6.8E-03 |
|  |  |  |  |  |  |
| **thickness** |  |  |  |  |  |
| term - preterm | -3.1E-02 | -4.3E-02 | -1.8E-02 | -4.8E+00 | <0.00001 |
| PC | -2.6E-02 | -2.7E-02 | -2.5E-02 | -4.8E+01 | <0.00001 |
| term:PC | 5.5E-03 | 3.4E-03 | 7.7E-03 | 5.1E+00 | <0.00001 |
| age | 1.9E-02 | 1.6E-02 | 2.1E-02 | 1.3E+01 | <0.00001 |
| male-female | -1.1E-02 | -2.0E-02 | -9.0E-04 | -2.2E+00 | 3.1E-02 |
|  |  |  |  |  |  |
| **FA** |  |  |  |  |  |
| term - preterm | 4.03E-03 | 2.09E-03 | 5.98E-03 | 4.06E+00 | <0.00001 |
| PC | -2.21E-03 | -2.42E-03 | -1.99E-03 | -1.98E+01 | <0.00001 |
| term:PC | -8.00E-04 | -1.27E-03 | -3.94E-04 | -3.73E+00 | 1.91E-04 |
| age | -9.40E-04 | -1.38E-03 | -5.04E-04 | -4.21E+00 | 3.20E-05 |
| male-female | -1.81E-03 | -3.32E-03 | -3.06E-04 | -2.36E+00 | 1.88E-02 |
|  |  |  |  |  |  |
| **MD** |  |  |  |  |  |
| term - preterm | -3.73E-05 | -4.65E-05 | -2.80E-05 | -7.89E+00 | <0.00001 |
| PC | 2.04E-05 | 1.98E-05 | 2.11E-05 | 6.10E+01 | <0.00001 |
| term:PC | -1.65E-06 | -2.96E-06 | -3.35E-07 | -2.46E+00 | 1.39E-02 |
| age | -5.14E-06 | -7.23E-06 | -3.05E-06 | -4.82E+00 | <0.00001 |
| male-female | 1.16E-05 | 4.41E-06 | 1.87E-05 | 3.17E+00 | 1.65E-03 |
|  |  |  |  |  |  |
| **ODI** |  |  |  |  |  |
| term - preterm | 1.75E-03 | -2.85E-03 | 6.35E-03 | 7.45E-01 | 4.57E-01 |
| PC | -8.56E-03 | 9.12E-03 | -8.01E-03 | -3.02E+01 | <0.00001 |
| term:PC | -9.43E-04 | -2.06E-03 | 1.70E-04 | -1.66E+00 | 9.69E-02 |
| age | 6.41E-03 | 5.37E-03 | 7.45E-03 | 1.21E+01 | <0.00001 |
| male-female | -5.91E-04 | -4.15E-03 | 7.45E-03 | -3.26E-01 | 7.45E-01 |
|  |  |  |  |  |  |
| **fICVF** |  |  |  |  |  |
| term - preterm | 4.40E-03 | 1.06E-03 | 7.74E-03 | 2.58E+00 | 1.02E-02 |
| PC | -7.59E-03 | -7.83E-03 | -7.36E-03 | -6.28E+01 | <0.00001 |
| term:PC | -2.38E-03 | -2.85E-03 | -1.90E-03 | -9.82E+00 | <0.00001 |
| age | 2.26E-03 | 1.50E-03 | 3.01E-03 | 5.86E+00 | <0.00001 |
| male-female | -5.56E-03 | -8.13E-03 | -2.98E-03 | -4.23E+00 | 3.01E-05 |
