# Supplementary material for: Cortical morphology at birth reflects spatiotemporal patterns of gene expression in the fetal human brain
Source: PLoS Biol. 2020 Nov 23;18(11):e3000976. doi: 10.1371/journal.pbio.3000976 (PMC7721147; doi:10.1371/journal.pbio.3000976)
Supplement: S8 Table — (DOCX) [file pbio.3000976.s019.docx]

## Table H: Estimated marginal means of each cortical metric for preterm and term cohorts

**S8 Table: Estimated marginal means of each cortical metric for preterm and term cohorts**

|  | **Estimated marginal means (S.D.)** | | |
| --- | --- | --- | --- |
| **metric** | **term** | **preterm** | **Effect size (Cohen's d)** |
| T1/T2 | 1.320 (0.0747) | 1.200 (0.0732) | 1.62 |
| thickness | 1.120 (0.0467) | 1.150 (0.0468) | -0.65 |
| FA | 0.130 (0.0073) | 0.126 (0.0072) | 0.56 |
| MD x 10^2^ | 0.112 (0.0034) | 0.116 (0.0034) | -1.17 |
| ODI | 0.387 (0.0171) | 0.385 (0.0170) | 0.12 |
| fICVF | 0.192 (0.0123) | 0.188 (0.0124) | 0.32 |
