# Supplementary material for: Cortical morphology at birth reflects spatiotemporal patterns of gene expression in the fetal human brain
Source: PLoS Biol. 2020 Nov 23;18(11):e3000976. doi: 10.1371/journal.pbio.3000976 (PMC7721147; doi:10.1371/journal.pbio.3000976)
Supplement: S9 Table — (DOCX) [file pbio.3000976.s020.docx]

**S9 Table: Cell class enrichments for differentially expressed genes in mouse model of fetal hypoxia**

|  | **All genes (n=15,502)** | | **Fetal gene markers (n=4733)** | |
| --- | --- | --- | --- | --- |
| **class**† | **enrichment ratio** | **p** | **enrichment ratio** | **p** |
| astrocyte | 3.96 | 0.021 | 1.21 | 0.433 |
| endothelial | 5.44 | <0.001 | 1.66 | 0.019 |
| microglia | 6.89 | <0.001 | 2.09 | 0.012 |
| neuron:excitatory | 0.89 | 0.662 | 0.27 | 0.999 |
| neuron:inhibitory | 0.68 | 0.770 | 0.21 | 0.991 |
| oligodendrocyte | 4.88 | <0.001 | 1.49 | 0.183 |
| OPC | 2.70 | 0.105 | 0.82 | 0.710 |
| pericyte | 4.88 | 0.027 | 1.48 | 0.340 |
| progenitor | 1.59 | 0.212 | 0.48 | 0.977 |
| radial glia | 2.23 | 0.017 | 0.68 | 0.924 |

†excluding neuron:unclassified
