# Supplementary material for: Cortical morphology at birth reflects spatiotemporal patterns of gene expression in the fetal human brain
Source: PLoS Biol. 2020 Nov 23;18(11):e3000976. doi: 10.1371/journal.pbio.3000976 (PMC7721147; doi:10.1371/journal.pbio.3000976)
Supplement: S10 Table — (DOCX) [file pbio.3000976.s021.docx]

**S10 Table: Enriched pathways within PPI networks in oligodendrocytes**

| **PPI network** | **Genes** | **Enriched Reactome pathways** | **Pathway ID** | **Pathway genes** | **FDR** |
| --- | --- | --- | --- | --- | --- |
| 1 | *ARHGEF10, ANKS1B, BCAR1, ENPP2, MOBP, OMG, PLLP, RHOB, SPATA2, TMTC4* | p75 NTR receptor-mediated signalling | HSA-193704 | *OMG, ARHGEF10* | 0.03050 |
|  |  | G alpha (12/13) signalling events | HSA-416482 | *RHOB, ARHGEF10* | 0.03050 |
|  |  | Rho GTPase cycle | HSA-194840 | *RHOB, ARHGEF10* | 0.03120 |
| 2 | *DLG1, CD200, GIPC1, GRIN2A, MARCH1, MAP1A* | Unblocking of NMDA receptors, glutamate binding and activation | HSA-438066 | *DLG1, GRIN2A* | 0.00049 |
|  |  | CREB phosphorylation through the activation of CaMKII | HSA-442729 | *DLG1, GRIN2A* | 0.00049 |
|  |  | Ras activation upon Ca2+ influx through NMDA receptor | HSA-442982 | *DLG1, GRIN2A* | 0.00049 |
|  |  | Synaptic adhesion-like molecules | HSA-8849932 | *DLG1, GRIN2A* | 0.00049 |
|  |  | RAF/MAP kinase cascade | HSA-5673001 | *DLG1, GRIN2A* | 0.00600 |
| 3 | *MPC1, PPR18, RPS6KA2, S100B, SERINC5, SFT2D1* | MyD88:MAL(TIRAP) cascade initiated on plasma membrane | HSA-166058 | *S100B, RPS6KA2* | 0.01810 |
|  |  | Toll Like Receptor 3 cascade | HSA-168164 | *S100B, RPS6KA2* | 0.01810 |
|  |  | TRIF(TICAM1)-mediated TLR4 signalling | HSA-937061 | *S100B, RPS6KA2* | 0.01810 |
|  |  | TRAF6 mediated induction of NFkB and MAP kinases upon TLR7/8 or 9 activation | HSA-975138 | *S100B, RPS6KA2* | 0.01810 |
|  |  | MyD88 cascade initiated on plasma membrane | HSA-975871 | *S100B, RPS6KA2* | 0.01810 |
|  |  | Signalling by interleukins | HSA-449147 | *S100B, RPS6KA2* | 0.02670 |
|  |  | Signalling by Receptor Tyrosine Kinases | HSA-9006934 | *S100B, RPS6KA2* | 0.02670 |
| 4 | *ACER3, FAIM2, NPC1, SMPD1* | Sphingolipid metabolism | HSA-428157 | *ACER3, SMPD1* | 0.00100 |
| 5 | *GOT1, CBR1* | - | - | - | - |
| 6 | *LDLRAD4, TSC22D4* | - | - | - | - |
| 7 | *UBSE2E2, SYT11, PTPRD* | - | - | - | - |
