# Supplementary material for: Cortical morphology at birth reflects spatiotemporal patterns of gene expression in the fetal human brain
Source: PLoS Biol. 2020 Nov 23;18(11):e3000976. doi: 10.1371/journal.pbio.3000976 (PMC7721147; doi:10.1371/journal.pbio.3000976)
Supplement: S11 Table — (DOCX) [file pbio.3000976.s022.docx]

**S11 Table: Enriched pathways within PPI networks in microglia**

| **PPI network** | **Genes** | **Enriched Reactome pathways** | **Pathway ID** | **Pathway genes** | **FDR** |
| --- | --- | --- | --- | --- | --- |
| 1 | *PTAFR, GNG5, LPAR6, GNG7, GPR183, ITGAX, IRF8, NFKB1, CYFIP1, PHACTR1, NCKAP1L* | G alpha (q) signalling events | HSA-416476 | *PTAFR, GNG5, GNG7, LPAR6* | 0.00036 |
|  |  | Neutrophil degranulation | HSA-6798695 | *PTAFR, ITGAX, NFKB1, CYFIP1, NCKAP1L* | 0.00036 |
|  |  | Rho GTPases activate WASPs and WAVEs | HSA-5663213 | *CYFIP1, NCKAP1L* | 0.00170 |
|  |  | Class A/1 (Rhodopsin-like receptors) | HSA-373076 | *PTAFR, LPAR6, GPR183* | 0.00220 |
|  |  | G alpha (i) signalling events | HSA-418594 | *GNG5, GNG7, GPR183* | 0.00340 |
|  |  | Interferon gamma signalling | HSA-877300 | *IRF8, PTAFR* | 0.00340 |
| 2 | *BARD1, H2AFX, CLSPN, H2AFZ, BRD2, PCNA, SIVA1, CKS2, CDCA3, CKS2, GADD45B, CDKN1A, PTP4A2, HIF1A, PMAIP1* | Cell cycle | HSA-1640170 | *H2AFX, H2AFZ, PCNA, BARD1, CLSPN, CDKN1A* | 0.00022 |
|  |  | Transcriptional regulation by TP53 | HSA-3700989 | *BARD1, PCNA, CDKN1A, PMAIP1* | 0.00130 |
|  |  | Cellular response to stress | HSA-2262752 | *H2AFX, H2AFZ, CDKN1A, HIF1A* | 0.00160 |
|  |  | Nonhomologous end-joining | HSA-5693571 | *H2AFX, BARD1* | 0.00390 |
|  |  | Deubiquitination | HSA-5688426 | *BARD1, CLSPN, HIF1A* | 0.00430 |
|  |  | Transcriptional regulation by RUNX3 | HSA-8878159 | *CDKN1A, BRD2* | 0.00650 |
|  |  | Apoptosis | HSA-109581 | *CLSPN, PMAIP1* | 0.01560 |
| 3 | *RHOB, CIT, ARHGAP25* | Rho GTPases activate CIT | HSA-5625900 | *RHOB, CIT* | 0.000065 |
|  |  | Rho GTPase cycle | HSA-194840 | *RHOB, ARHGAP25* | 0.001000 |
| 4 | *RPS27L, RPS27, EEF2* | Eukaryotic translation elongation | HSA-156842 | *RPS27L, RPS27, EEF2* | 0.0000056 |
| 5 | *RAB7A, TGOLN2* | Membrane trafficking | HSA-199991 | *RAB7A, TGOLN2* | 0.0216 |
| 6 | *SON, PRR2C* | - | - | - | - |
| 7 | *JUNB, TRIB1* | - | - | - | - |
| 8 | *DNAJB11, HSPH1* | - | - | - | - |
| 9 | *NR4A1, PPP3CA, MEF2A* | - | - | - | - |
| 10 | *YWHAB, MAP3K2* | - | - | - | - |
