# Supplementary material for: Cortical morphology at birth reflects spatiotemporal patterns of gene expression in the fetal human brain
Source: PLoS Biol. 2020 Nov 23;18(11):e3000976. doi: 10.1371/journal.pbio.3000976 (PMC7721147; doi:10.1371/journal.pbio.3000976)
Supplement: S12 Table — (DOCX) [file pbio.3000976.s023.docx]

**S12 Table: Enriched pathways within PPI networks in endothelial cells**

| **PPI network** | **Genes** | **Enriched Reactome pathways** | **Pathway ID** | **Pathway genes** | **FDR** |  |
| --- | --- | --- | --- | --- | --- | --- |
| 1 | *CASP3, TGM2, CDKN1A, PMAIP1, GADD45A, GADD45B, PTP4A2, CCNT1, CCNY, TAF13, EIF4H, RPS27, HNRNPA0, SRRM1, BCL2L11, YWHAB, TBC1D4, FOXO1, DYRK1A, CSNK1A1, SGK1, RAB7A, ARF6, GIT2* | Intrinsic pathway for apoptosis | HSA-109606 | *CASP3, BCL2L11, PMAIP1, YWHAB* | 0.00005 |  |
|  |  | Transcriptional regulation by TP53 | HSA-3700989 | *SGK1, YWHAB, PMAIP1, CDKN1A, GADD45A, CCN1, TAF13* | 0.00005 |  |
|  |  | Constitutive signalling by AKT1 E17k in cancer | HSA-5674400 | *CDKN1A, FOXO1* | 0.01050 |  |
|  |  | TBC/RABGAPs | HSA-8854214 | *RAB7A, ARF6* | 0.02510 |  |
|  |  | TP53 regulates transcription of cell cycle genes | HSA-6791312 | *CDKN1A, GADD45A* | 0.03010 |  |
|  |  | Translation initiation complex formation | HSA-72649 | *EIF4H, RPS27* | 0.03510 |  |
| 2 | *GNG5, KCNJ2, RAMP2, LPAR6, CXCL1* | GPCR ligand binding | HSA-500792 | *GNG5, RAMP2, LPAR6, CXCL1* | 0.00009 |  |
|  |  | Activation of G protein gated potassium channels | HSA-1296041 | *GNG5, KCNJ2* | 0.00064 |  |
| 3 | *MRPL57, MRPL33* | Mitochondrial translation initiation | HSA-5368286 | *MRPL57, MRPL33* | 0.00013 |  |
|  |  |  |  |  |  |  |
| 4 | *RHOB, ARHGAP18, ARHGEF10, PPP1R12A, MYH10* | Signalling by Rho GTPases | HSA-194315 | *RHOB, ARHGAP18, ARHGEF10, PPP1R12A, MYH10* | 0.0000001 |  |
|  |  | Rho GTPases activate PAKs | HSA-5627123 | *PPP1R12A, MYH10* | 0.0000640 |  |
|  |  | Sema4D induced cell migration | HSA-416572 | *RHOB, MYH10* | 0.0000640 |  |
|  |  | G alpha (12/13) signalling events | HSA-416482 | *RHOB, ARHGEF10* | 0.0044000 |  |
| 5 | *ATP11A, ATP1B2* | Ion transport by P-type APTases | HSA-936837 | *ATP11A, ATP1B2* | 0.000090 |  |
| 6 | *SOCS3, RNF19A, UBR2* | Ubiquitination & proteasome degradation | HSA-983168 | *SOCS3, RNF19A, UBR2* | 0.000110 |  |
| 7 | *MAML2, TLE1* | NOTCH1 intracellular domain regulates transcription | HSA-2122947 | *MAML2, TLE1* | 0.000190 |  |
| 8 | *SNRK, DUSP5* | - | - | - | - |  |
|  | | | | | | |
